# Imputation in the Wild: Genome‐Wide Robustness and Fine‐Scale Limitations of Low‐Coverage Genomes in Endangered Species

**DOI:** 10.1111/1755-0998.70179

**Published:** 2026-07-27

**Authors:** Lucía Mayor‐Fidalgo, Enrico Bazzicalupo, Laia Pérez‐Sorribes, Laura Soriano, José A. Godoy

**Affiliations:** ^1^ Departamento de Ecología y Evolución Estación Biológica de Doñana, Consejo Superior de Investigaciones Científicas Sevilla Spain; ^2^ Department of Population Analysis and Monitoring Swedish Museum of Natural History Stockholm Sweden

**Keywords:** demographic history, genotype imputation, inbreeding, local ancestry inference, low‐coverage whole‐genome sequencing

## Abstract

Low‐coverage whole‐genome sequencing (lcWGS) combined with genotype imputation is increasingly being used to generate large genomic datasets at reduced cost, offering a promising alternative for conservation genomics. Here, we use the Iberian lynx ( *Lynx pardinus* ), a species with extremely low genetic diversity, genetically differentiated populations, and recent admixture, as a case study to evaluate the performance of imputed lcWGS data for population genomic inferences. We performed a leave‐one‐out imputation approach with 50 individuals sequenced to high coverage to assess imputation accuracy under different reference panel compositions, sequencing depths, and imputation strategies. We then compared inbreeding, admixture, and demographic history inferences derived from high‐coverage and imputed datasets under highly concordant imputation scenarios (*r* ^2^ > 0.98; NRD rate < 0.05). Imputation accuracy substantially improved when using a mixed‐ancestry reference panel, a multi‐target imputation setting, and an adjusted effective population size parameter, particularly at low sequencing coverages. Genome‐wide estimates of inbreeding and ancestry were largely robust. In contrast, fine‐scale local genomic patterns were more sensitive to lower levels of imputation accuracy: long runs of homozygosity and ancestry tracts were reliably recovered, whereas shorter segments, which reflect older demographic events, were more error‐prone. Consistent with these patterns, recent demographic trajectories were accurately reconstructed, while older effective population size estimates showed greater uncertainty. Overall, our results highlight the importance of validating and optimizing imputation performance in species with complex demographic histories and support lcWGS followed by imputation as a reliable framework for the study of recent demographic events in endangered species.

## 1 Introduction

Advances in sequencing technology and the associated decline in per‐base sequencing costs have made whole‐genome approaches increasingly feasible. Still, high‐coverage whole‐genome sequencing (WGS) remains prohibitively expensive for many conservation efforts targeting endangered species. As an alternative, low‐coverage WGS (lcWGS) provides an affordable framework to capture genome‐wide variation, at the cost of lower reliability (Watowich et al. 2023). Probabilistic methods have been developed to integrate the genotype uncertainty associated with lcWGS data in the estimation of population‐level genetic parameters such as allele frequencies, linkage disequilibrium (LD), and population structure (Lou et al. 2021). Another strategy is to improve genotype calling accuracy through imputation methods, which use LD patterns and haplotypes observed in a smaller high‐confidence reference dataset to infer the missing genotype data (Lou et al. 2021; Watowich et al. 2023).

The reduced costs of lcWGS followed by imputation opens the door to a wide range of precise genomic analyses using large sample sizes, offering an improved resolution and sensitivity in demographic reconstructions, deleterious variants detection, or inbreeding and admixture characterization (Fuentes‐Pardo and Ruzzante 2017; McLaughlin and Winker 2020; Supple and Shapiro 2018). This strategy has already been used to obtain genome‐wide data from a large number of individuals in farmed (Liu et al. 2024; Teng et al. 2022; Yang et al. 2024), domestic (Buckley et al. 2022; Kaelin et al. 2024; Wragg et al. 2024), and increasingly in wild species (Freudiger et al. 2025; Fuller et al. 2020; Hewett et al. 2025; Puckett et al. 2023).

Imputation accuracy is determined by multiple interacting factors. At a genome‐wide level, increasing sequencing depth reduces genotype uncertainty. Imputation performance can also be affected by allele frequency and variant type, with common SNPs typically imputed more reliably than rare variants or indels (Marchini and Howie 2010; Nguyen et al. 2024). The properties of the reference panel are equally critical: its size, haplotype diversity, ancestry composition, and relatedness to the target population shape imputation outcomes (Lloret‐Villas et al. 2023; Rowan et al. 2019; Vi et al. 2025; Wragg et al. 2024). In addition, analytical choices, such as phasing strategies and imputation parameterization, can further contribute to imputation performance (Jiang et al. 2022; Pook et al. 2020; Sun et al. 2025). Locally, accuracy is modulated by patterns of LD (He et al. 2015; Hickey et al. 2012; Lau et al. 2024; Yang et al. 2024), which are population‐specific and can vary across the genome (Goddard and Hayes 2012; Slatkin 2008; Zavattari et al. 2000). For instance, regions of high recombination often show reduced performance because haplotype blocks are shorter and LD decays more rapidly (Hanks et al. 2022; Nguyen et al. 2024; Weng et al. 2014). Given the combined influence of all these factors, empirical evaluation of reference panel performance is a key step to ensure reliability when using imputed genomes in downstream analyses.

Many studies have evaluated the accuracy of genotype imputation by applying leave‐one‐out validation approaches, whereby a single individual is sequentially removed from the reference panel, downsampled to simulate lcWGS data, and imputed based on the remaining samples (Flanagan et al. 2024; Heidaritabar et al. 2022; Li et al. 2021; Ni et al. 2015). This design provides an unbiased estimate of imputation accuracy since the target individual does not contribute haplotype information to its own imputation and the reference panel composition remains similar. While widely applied in humans and livestock with large reference panels, applications in wild species remain scarce, generally showing adequate but variable imputation accuracy scores (Enbody et al. 2023; Larison et al. 2021; Vi et al. 2025; Wu et al. 2024).

Importantly, relatively few studies have comprehensively evaluated the impact of imputed genotypes on fine‐scale genomic features, which can be especially vulnerable to localized genotype errors, where a few misimputed sites may lead to spurious inferences. These examples primarily focused on identity‐by‐descent segments in systems with large and diverse reference panels, including humans (Sousa da Mota et al. 2023), macaques (Freudiger et al. 2025), and canids (Bougiouri et al. 2025), revealing deviations in accuracy that depended on coverage and tract length. In conservation contexts, the evidence is even more limited, where only a small number of studies have assessed how imputed genotypes affect downstream genomic inferences relevant for natural populations. These investigations have generally focused on genome‐wide metrics and have shown that imputed data can recover key parameters reliably even when using relatively small reference panels: Watowich et al. (2023) estimated population structure and relatedness in 68 gelada monkeys, and Tan et al. (2025) examined effects on heterozygosity, inbreeding and the site‐frequency spectrum in 30 hihi individuals. However, because these analyses were restricted to genome‐wide summaries, it remains unclear whether biases similar to those detected in other systems arise when using small reference panels in populations with low genetic diversity and strong population structure, conditions typical of many conservation‐relevant species.

In this study, we used the Iberian lynx ( *Lynx pardinus* , Temminck 1827) as a model system due to its distinctive demographic history. The species has undergone recurrent bottlenecks and long‐term small population sizes, leading to concerning levels of genetic erosion and a high differentiation between the two populations that survived its most recent decline (Abascal et al. 2016; Casas‐Marce et al. 2013; Kleinman‐Ruiz et al. 2022; Lucena‐Perez et al. 2021). The Doñana population has maintained very small effective sizes for decades (Casas‐Marce et al. 2017; Godoy et al. 2025), resulting in extreme loss of genetic diversity (Lucena‐Perez et al. 2024), high inbreeding and LD extending over long distances (Abascal et al. 2016). In contrast, the Andújar‐Cardeña population retained comparatively higher diversity and larger effective sizes until the most recent bottleneck (Casas‐Marce et al. 2017; Godoy et al. 2025). In addition, ongoing conservation management has generated admixed individuals that combine ancestry from both populations through translocations and captive breeding (Kleinman‐Ruiz et al. 2017, 2019). Together, these three groups provide a unique framework to test imputation performance in a classical conservation genomics context, allowing the comparison between the strongly bottlenecked Doñana population (BT), the less bottlenecked Andújar‐Cardeña (NBT), and their admixed descendants (MIX).

We evaluate genotype imputation performance in the different populations of Iberian lynx using a leave‐one‐out approach. First, we begin by assessing imputation performance under alternative reference panel compositions (population‐specific versus mixed ancestries), imputation designs (imputing each sample independently versus multiple samples simultaneously), and phasing and imputation parameters. We then examine how imputed datasets with varying levels of imputation accuracy perform relative to high‐coverage data across a range of downstream genomic analyses relevant for species that have experienced severe population declines followed by a recovery through admixture. These include both genome‐wide genetic estimates, such as inbreeding coefficients and global ancestry proportions, and fine‐scale genomic inferences that rely on the contiguity of accurately imputed genotypes, namely runs of homozygosity (ROH) calling, local ancestry inference, and demographic history reconstruction based on LD. Together, these analyses provide a practical framework for the use of imputed data in small, structured, and demographically complex populations characteristic of endangered species.

## 2 Material and Methods

### 2.1 WGS Data Generation and Processing

#### 2.1.1 Sampling, DNA Extraction and Sequencing

We generated new whole‐genome high‐coverage resequencing data for 50 Iberian lynxes to conform a reference panel of high‐confidence haplotypes for the imputation of lcWGS data. These samples were chosen with the aim of capturing the maximum diversity of haplotypes existing in the current populations: the most strongly bottlenecked Doñana population (BT, *n* = 15), the more diverse and less bottlenecked Andújar‐Cardeña population (NBT, *n* = 14), and individuals born from their admixture (MIX, *n* = 21). Therefore, we selected individuals that: (1) had a high number of descendants, (2) did not constitute complete parent‐offspring trios within the reference panel, and (3) belonged to the different populations. Hence, the reference panel was composed of individuals spanning a range of ancestry backgrounds, which were reflected by the pedigree‐based ancestry coefficient *θₛ* (see Table S1), ranging from 0 (individuals with pure BT ancestry) to 1 (pure NBT ancestry). Because close relatives within a reference panel may contribute to increased imputation accuracy, pairwise relatedness among individuals was estimated using KING (Manichaikul et al. 2010). Most individuals were unrelated or distantly related, with only 1.2% of pairs considered as first‐degree relatives, 6.1% as second‐degree, and 6.7% as third‐degree, according to KING standard thresholds (Figure S1).

DNA was extracted from tissue or blood using the Maxwell 16 LEV Blood DNA Kit (Qiagen, Valencia, CA, USA) and quantified using the Qubit dsDNA HS Assay kit (Thermo Fisher Scientific). Samples yielding too low DNA concentrations were re‐extracted using classical phenol‐chloroform methodologies. gDNA extracts were then sent for paired‐end library preparation and sequencing to a target depth of 30× on the Illumina NovaSeq X Plus platform at Novogene Ltd. facilities (Cambridge, UK).

To test how imputation performance benefits from the simultaneous imputation of multiple samples (multi‐target imputation assay, see below), available WGS data from 20 individuals sequenced at medium coverage (5–6×) and 11 sequenced at high‐coverage (21–30×) during previous projects (Abascal et al. 2016; Lucena‐Perez et al. 2021) were included as additional targets and jointly imputed alongside the focal leave‐one‐out sample. These samples were not included in the reference panel because they differed from the newly generated dataset in sequencing coverage and quality (Kleinman‐Ruiz et al. 2022). Additionally, we incorporated 168 low‐coverage (0.05–3×) newly sequenced samples. Their DNA was extracted using the same methodologies described above, and genomic libraries were prepared with the Nextera XT Illumina library preparation kit, following a miniaturized protocol where reaction volumes were reduced to ~25%. Libraries were also sent for sequencing on Illumina NovaSeq X Plus platform at Novogene Ltd. facilities (Cambridge, UK).

#### 2.1.2 Reads Processing and Alignment

For all samples, resequencing data processing followed the default Illumina analysis pipeline. Sequencing reads were pre‐processed using fastp (Chen 2023), applying base correction based on overlapping paired‐end reads, removing adapter sequences and poly‐G tails, and retaining only those reads with a minimum post‐trimming length of 30 base pairs. Quality assessment of resulting reads was performed with FastQC (Andrews 2010) and multiQC (Ewels et al. 2016). Sequencing reads were then aligned to the new version of the Iberian lynx reference genome (mLynPar1.2, https://denovo.cnag.cat/pardinus_data) using BWA‐MEM (Li 2013). Following alignment, duplicate reads were identified and flagged using the MarkDuplicates tool from the Picard software suite (https://broadinstitute.github.io/picard). To improve alignment accuracy around INDEL regions, realignment was conducted with GATK's RealignerTargetCreator and IndelRealigner tools (McKenna et al. 2010). We evaluated the quality of the alignments and estimated the average sequencing depth per sample using Qualimap2 (Okonechnikov et al. 2016). Table S1 lists details regarding each sample's *θₛ*, sequencing batch and average sequencing depth obtained.

#### 2.1.3 Reference Panel Preparation: Variant Calling, Filtering and Phasing

For the reference panel VCF generation, variants were called using the alignments of the 50 samples resequenced at high coverage for this study, using DeepVariant v1.6 (Poplin et al. 2018) with the *WGS Illumina*‐trained model. This produced one gVCF output per sample, which was merged with the *DeepVariantWGS* configuration in GLnexus v1.4.1 (Yun et al. 2021), resulting in a set of 4,288,231 variants.

Low complexity regions and interspersed repeats in the reference genome were annotated using a combination of RepeatModeler and RepeatMasker (http://www.repeatmasker.org). Variants falling within these regions were excluded from the VCF, along with all INDELs, non‐biallelic sites, invariant positions, and variants with low quality scores (QUAL < 20).

To account for potential reference genome regions where multiple paralogs may have collapsed, we estimated mean sequencing depth in non‐overlapping 10 kb windows across the genome with mosdepth (Pedersen and Quinlan 2018). We excluded windows where the total depth exceeded the population mean by more than 0.5 standard deviations. Lastly, we removed SNPs with missing data in more than 2% of the samples. After keeping only the variants found in the scaffolds of the reference genome assigned to one of the 18 autosomes, our final reference panel consisted of 1,259,114 SNPs.

As imputation requires phased genotypes, we employed a two‐step statistical phasing strategy. First, we used WhatsHap v1.1 (Martin et al. 2016) to generate phase sets using the *‐‐tag = PS* option. Next, we applied SHAPEIT v4.2.1 (Delaneau et al. 2019) to infer haplotypes for each individual based on the phase set information. Since no specific linkage map is available for the Iberian lynx, we applied a homogeneous recombination rate equal to the domestic cat average of 1.9 cM/Mbp (Li et al. 2016), an approach previously adopted in multiple feline studies (Figueiró et al. 2017; Lan et al. 2025; Li et al. 2019; Westbury et al. 2021). The phasing was initially run with an expected genotyping error rate of 0.0001 and the MCMC scheme ‘10b,1p,1b,1p,1b,1p,1b,1p,10m’, as recommended in the SHAPEIT4 documentation.

### 2.2 Leave‐One‐Out Imputation Framework

#### 2.2.1 Imputation Scenarios and Experimental Design

To simulate lcWGS data, we randomly downsampled the BAM files of the 50 individuals from the reference panel to six target coverages (0.1×, 0.25×, 0.5×, 1×, 1.5×, and 2×) using samtools v1.19 (Li et al. 2009). Genotype likelihoods at reference panel variant sites were then computed for each downsampled sample with bcftools *mpileup* and *call* (Li et al. 2009).

**FIGURE 1:**
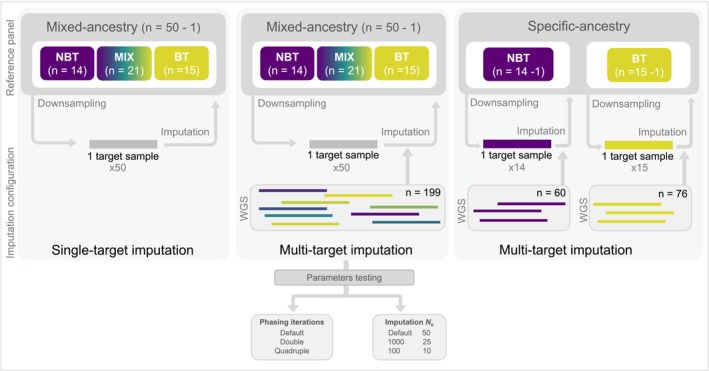
Schematic figure of the three leave‐one‐out imputation assays performed. The top grey boxes represent the two different reference panel compositions tested. The mixed‐ancestry reference panel (left and center) includes all high‐coverage sequenced individuals from the different populations: the bottlenecked (BT), the non‐bottlenecked (NBT) and the admixed one (MIX). In the third assay (right), BT and NBT samples were imputed using a specific‐ancestry reference panel. Two imputation configurations were evaluated: single‐target imputation (left panel), in which only the downsampled reference panel individual was imputed; and multi‐target imputation (center and right), in which the downsampled individual was imputed jointly with additional whole‐genome sequenced target samples with matching ancestral background. Different values of phasing iterations and imputation effective population sizes (*N*_e_) were tested under the multi‐target configuration with a mixed‐ancestry reference panel imputation assay.

Imputation was performed using GLIMPSE v1 (Rubinacci et al. 2021) following a leave‐one‐out strategy, whereby each downsampled individual was excluded from the reference panel used for its imputation. To evaluate the effects of reference panel composition and simultaneous imputation of multiple target samples, we conducted three different imputation assays (Figure 1). First, we performed single‐target imputation using a mixed‐ancestry reference panel, composed of all individuals (excluding the target) and imputing each downsampled individual independently. Second, we performed multi‐target imputation using the same mixed‐ancestry reference panel, but imputing each downsampled individual jointly with additional target samples belonging to all coverage groups (*n* = 199). Third, to assess the effect of ancestry‐matched reference panels, we imputed individuals from the BT (*n* = 15; 514,039 SNPs) and NBT (*n* = 14; 1,079,599 SNPs) populations using reference panels composed only of individuals of the same pure ancestry. Given that multi‐target imputation consistently outperformed single‐target imputation in the previous assays (see Section 3), these specific‐ancestry reference panels were evaluated exclusively under the multi‐target imputation framework, utilizing additional target WGS samples from the corresponding ancestry groups. All these assays were initially performed using GLIMPSE1 default parameters.

We then used the best‐performing configuration (multi‐target imputation with the mixed‐ancestry reference panel; see Section 3) to evaluate the effects of changing key phasing and imputation parameters. First, we varied the number of iterations in the MCMC scheme implemented in SHAPEIT4, testing three settings: the recommended configuration (‘10b,1p,1b,1p,1b,1p,1b,1p,10m’), a doubled iteration scheme (‘20b,2p,2b,2p,2b,2p,2b,2p,20m’), and a quadrupled iteration scheme (‘40b,4p,4b,4p,4b,4p,4b,4p,40m’). Second, we explored the effect of the effective population size parameter (−ne) in GLIMPSE_phase by testing a range of values: default (20,000), 1000, 100, 50, 25, and 10. All imputation assays performed are summarized in Figure 1.

#### 2.2.2 Imputation Accuracy Assessment

Imputation accuracy was evaluated using several metrics at different levels of resolution: genome‐wide, across minor allele frequency (MAF) bins, and across samples. First, we computed the global squared Pearson correlation coefficient (*r* ^2^) between the dosage values of imputed and high‐coverage genotypes, which measures the overall agreement between imputed and true genotypes, together with the non‐reference discordance (NRD) rate, which quantifies errors specifically among non‐reference genotypes, using bcftools *stats*. Second, we estimated the Imputation Quality Score (IQS) (Lin et al. 2010), which is particularly informative for low‐frequency variants, and the *r* ^2^ across MAF bins, computed with the Python script available at https://github.com/TorkamaniLab/imputation_accuracy_calculator (Chen et al. 2020) and with GLIMPSE_concordance, respectively. Finally, we assessed sample‐level accuracy by calculating each individual's genotype concordance as the proportion of correctly imputed genotypes, including a specific estimate for heterozygous calls.

To assess the effects of SNP imputation quality on accuracy, we compared the obtained *r* ^2^ estimates across variants stratified by two imputation quality metrics produced by GLIMPSE1. Specifically, we evaluated different INFO scores, a variant‐level metric that measures the relative statistical information in the imputed genotypes probabilities compared with perfectly observed genotypes (Marchini and Howie 2010); and the maximum genotype probability (maxGP), defined as the highest posterior probability among the three possible genotypes for a given SNP and individual, providing an individual‐level measure of confidence in the imputed genotype call (Rubinacci et al. 2021).

### 2.3 Population Genomic Estimations Comparison From High‐Coverage and Imputed Data

To evaluate the impact of imputation accuracy on downstream population genomic inferences of inbreeding, admixture, and demographic history, we performed all analyses on the 50 individuals of the reference panel using both the high‐coverage WGS data and the imputed genotypes datasets covering different levels of imputation performance. These datasets were grouped into four classes (R1–R4, from lower to higher accuracy; Table 1), selected from imputation conditions showing high accuracy values (*r* ^2^ > 0.98; NRD rate < 0.05) in preliminary leave‐one‐out validation analyses. This setup represents a range of reliability scenarios for downstream applications, allowing us to assess whether subtle differences in imputation quality could still influence genomic inferences. All selected datasets were generated using the imputation configuration that achieved the best overall performance (multi‐target imputation using a mixed‐ancestry reference panel; see Section 3) across several sequencing coverages and –ne parameter values (Table 1).

**TABLE 1:** Definition of accuracy levels used for downstream population genomic analyses.

Accuracy level	Sequencing coverage	GLIMPSE_phase –ne value	*r* ^2^	NRD rate
R1	0.5×	Default (20,000)	0.984	0.028
R2	1×	Default (20,000)	0.991	0.014
R3	0.1×	10	0.992	0.012
R2	0.5×	10	0.996	0.006

*Note:* Different combinations of sequencing coverage and GLIMPSE_phase (−ne) parameter settings were selected to cover a range of imputation accuracies, measured as overall genotype correlation coefficient (*r* ^2^) and non‐reference discordance (NRD) rate.

To assess the agreement between imputed and high‐coverage estimates, we used different comparison strategies depending on the genomic resolution of the analysis. For genome‐wide summary metrics—inbreeding coefficients based on ROH (*F*_ROH_), ancestry proportions and effective population sizes—discrepancies between imputed and high‐coverage estimates were summarized using the median of the differences and standardized effect sizes via Cohen's *d* (*d*), computed using the R package *effsize* (Torchiano 2016). In contrast, for fine‐scale results based on genomic segments (i.e., ROH and local ancestry analyses), agreement was assessed using overlap‐based metrics. First, we calculated the fraction of overlap of each imputed segment with any high‐coverage segment belonging to the same class—defined as either a ROH or a specific ancestry state—using BEDTools *coverage* (Quinlan and Hall 2010). We additionally computed the inverse overlap, by estimating the fraction of overlap of each high‐coverage segment with any imputed segment of the same class. Overlap fractions were then averaged across segments within each individual for each class, tract length bin, and coverage level. Finally, an imputed segment was classified as overlapping if it shared at least 75% of its length with any high‐coverage segment of the same class, while segments with less than 75% overlap were classified as non‐overlapping. All results were visualized using the R package *ggplot2* (Wickham 2016).

#### 2.3.1 Recent Demographic History Reconstruction

Demographic history over the past 100 generations was inferred for the BT (*n* = 14, one individual excluded for being the parent of another one) and the NBT (*n* = 14) populations. We used the software GONE (Santiago et al. 2020), which estimates recent *N*_*e*_ based on patterns of LD. Input files were prepared using PLINK v1.9 (Purcell et al. 2007). For each dataset, we ran 50 independent replicates using standard GONE parameters, but changing the average recombination rate to that of the domestic cat (1.9 cM/Mbp) and the number of SNPs used per replicate to 15,000 SNPs per chromosome, to reflect the relatively low SNP density in the Iberian lynx genome and to generate enough independent replicates.

#### 2.3.2 Inbreeding Characterization

ROH were inferred using the Hidden Markov Model (HMM) approach implemented in the R package *RZooRoH* v0.3.2.1 (Bertrand et al. 2019; Druet and Gautier 2017, 2022), which models individual genomes as mosaics of homozygous‐by‐descent (HBD) and non‐HBD segments. RZooRoH has the advantages of accounting for genotype uncertainty (using genotype probabilities) and not requiring specific parameter adjustment for ROH calling like rule‐based methods. The mixKR model was applied independently to each population, specifying 14 HBD classes and a single non‐HBD class. Each HBD class is associated with a rate parameter *R*_*k*_, which is inversely related to the time since the common ancestor (*R*_*k*_ ≈ 2 × g, where g is the expected number of generations since the inbreeding event). In our model, we used R_k_ values in powers of 2 (2, 4, 8, …) up to 16,384. Physical genomic coordinates were converted to genetic positions assuming a uniform recombination rate of 1.9 cM/Mbp. For the high‐coverage dataset, ROH were estimated using hard genotype calls, while the imputed datasets were analysed using the genotype probabilities generated by GLIMPSE1, in order to account for genotype uncertainty associated with the imputation process.

Global inbreeding coefficients (*F*_ROH_) were estimated by integrating the HBD probabilities using the function *cumhbd* across HBD classes to capture the contribution of inbreeding events occurring at different time scales. Additionally, to assess the accuracy of ROH called from imputed data, we computed the reciprocal overlap between the imputed and the high‐coverage datasets across ROH length bins of 0.5–1, 1–2, 2–4, 4–8, 8–16, 16–32, and 32+ Mbp.

Since RZooRoH provides an estimate of the probability that each SNP belongs to a HBD‐segment, we calculated the average HBD probability of all markers of each inferred ROH as a proxy of its inference uncertainty using the function *probhbd*, setting the threshold of HBD contribution up to class T = 512. We then explored the impact that different mean HBD‐probability thresholds (0.8, 0.9, and 0.95) would have on the number of overlapping ROH (retention of true positives) and non‐overlapping ROH (elimination of false negatives).

#### 2.3.3 Genome‐Wide and Local Ancestry Inference

Global ancestry proportions were estimated using ADMIXTURE v1.3 (Alexander et al. 2009) with standard parameters and K = 2, and an LD‐pruning performed with PLINK v1.9. To characterize local ancestry patterns, we used the software RFMix v2 (Maples et al. 2013), which uses a conditional random field (CRF) model to assign ancestry along query haplotypes based on a panel of reference haplotypes observed in individuals of pure ancestry. Ancestry is estimated at predefined CRF points, which represent genomic intervals of assumed homogeneous ancestry. Our reference set of haplotypes only comprised the high‐coverage phased genotypes of pure ancestry individuals from the BT (*n* = 15) and NBT (*n* = 14) populations. Query haplotypes consisted of the 21 MIX individuals, both in the high‐coverage and the imputed datasets. The same genetic map file used for phasing was provided to RFMix2.

Similar to the ROH analysis, we computed the reciprocal fraction of overlap between high‐coverage and imputed datasets for local ancestry tracts. This was done separately for each ancestry category: homozygous BT (BT‐BT), heterozygous (BT‐NBT) and homozygous NBT (NBT‐NBT) across several tract length bins (< 0.1, 0.1–0.5, 0.5–1, 1–2, 2–4, 4–8, 8–16, 16–32, 32–64, 64–128 and 128+ Mbp). We also computed individual confusion matrices to compare the proportion of SNPs assigned to each ancestry category per sample in the high‐coverage and the imputed datasets and to assess the directionality of deviations. Confusion matrices were then averaged across individuals, grouped by their pedigree‐expected ancestry proportions (*θₛ*): mostly BT (0 < *θₛ* < 0.5), mostly NBT (0.5 < *θₛ* < 1) and approximately equal BT‐NBT ancestry (*θₛ* = 0.5).

Finally, we calculated ancestry assignment uncertainty of each ancestry tract as the average marginal posterior probability of the most probable ancestry of the CRF points overlapping that tract. We then evaluated how applying different minimum probability thresholds (0.9, 0.95, and 0.99) affected the rate of overlapping and non‐overlapping ancestry tracts retained.

## 3 Results

### 3.1 Imputation Accuracy and Filtering Strategies

**FIGURE 2:**
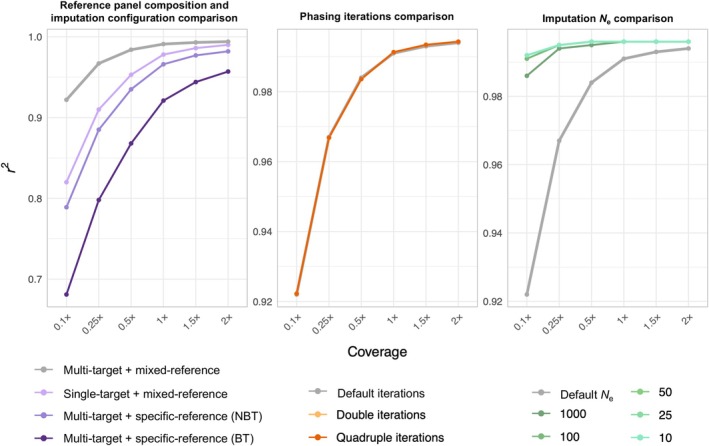
Global imputation accuracy, measured as *r* ^2^, across imputation assays. Left panel summarizes *r* ^2^ values obtained under alternative reference panel compositions—either a mixed‐reference (including all high‐coverage sequenced individuals) or a specific‐reference (including only individuals from the bottlenecked, BT, or non‐bottlenecked, NBT, populations)—and different imputation configurations, namely single‐target imputation (only the downsampled individual was imputed) and multi‐target imputation (the downsampled individual was imputed jointly with additional whole‐genome sequenced samples sharing the same ancestral background). The center panel shows *r* ^2^ values for the multi‐target, mixed‐reference imputation assay (which yielded the highest overall accuracy) across different settings of the SHAPEIT –mcmc parameter (phasing iterations). The right panel displays *r* ^2^ values for the same assay under varying effective population sizes (GLIMPSE_phase –ne parameter) during imputation. Increasing the number of phasing iterations did not lead to further improvements in imputation accuracy, whereas reducing the effective population size resulted in a substantial increase in *r* ^2^.

Across all imputation scenarios, multi‐target imputation using the mixed‐ancestry reference panel consistently yielded the highest imputation accuracy, especially at coverages under 0.5× (Figure 2 and Figure S2). Single‐target imputation using the mixed‐ancestry reference panel showed reduced accuracy across all coverages, with the largest differences observed at low sequencing depths (e.g., *r* ^2^ = 0.82 at 0.1× with single‐target vs. *r* ^2^ = 0.92 with multi‐target), indicating GLIMPSE1 highly benefits from the imputation of multiple target samples at the same time. The smaller ancestry‐specific reference panels, evaluated exclusively under the multi‐target framework, also resulted in consistently lower *r* ^2^ and higher NRD rates than the mixed‐ancestry multi‐target baseline, with a more pronounced reduction in the BT population. For instance, at 0.5× coverage, *r* ^2^ increased from 0.86 with the BT‐specific reference panel to 0.97 when using the mixed‐ancestry reference panel. Overall, these results show that simultaneous imputation of multiple samples using a larger mixed‐ancestry reference panel maximizes imputation accuracy across the tested conditions, and was therefore the approach used for downstream analyses.

Adjusting the –ne parameter during imputation substantially improved performance, with progressively smaller *N*_e_ values yielding consistently higher accuracy compared to the default setting of *N*_e_ = 20,000 (Figure 2 and Figure S2). Notably, 0.1× coverage imputed with a *N*_e_ = 10 already achieved a slightly higher imputation accuracy (*r* ^2^ = 0.992; NRD rate = 0.012) than 1× coverage imputed with the default *N*_e_ (*r* ^2^ = 0.991; NRD rate = 0.014). In contrast, increasing the number of iterations during the phasing step did not substantially improve imputation performance, with nearly identical *r* ^2^ values and NRD rates across settings (e.g., at 0.1× coverage, *r* ^2^ was 0.9223 under all three iteration schemes, while NRD rate ranged only from 0.1501 to 0.1503; Figure 2 and Figure S2).

The positive effect on accuracy of using an adjusted *N*_e_ value of 10 was consistently observed across allele frequency classes (Figure S3) and individuals (Figure S4), particularly at lower coverage levels. Across MAF bins, imputation performed with *N*_e_ = 10 achieved mean *r* ^2^ values above 0.80 for variants with MAF < 0.02 already at 0.1× coverage, compared to *r* ^2^ = 0.62 when using default *N*_e_ (Figure S3). At the sample level, coverage increased concordance rates under both parameterizations, although gains became marginal beyond 0.5× when using *N*_e_ = 10, and beyond 1.5× under default setting (Figure S4). Heterozygous sites concordance remained consistently lower regardless of parameter choice, although when using *N*_e_ = 10, heterozygous concordance reached 97% at 0.1×, compared to ~75% using the default value.

The application of INFO scores and maxGP filtering thresholds led to consistent improvements in accuracy (Figures S5 and S6) when imputation performance was poorer (default *N*_e_), at the cost of introducing more missing genotypes. When using an adjusted *N*_e_ during imputation, however, filtering led to almost no improvement, as concordance values were already high, and increases in accuracy were only detectable at very low MAF bins.

### 3.2 Recent Demographic History Inference

**FIGURE 3:**
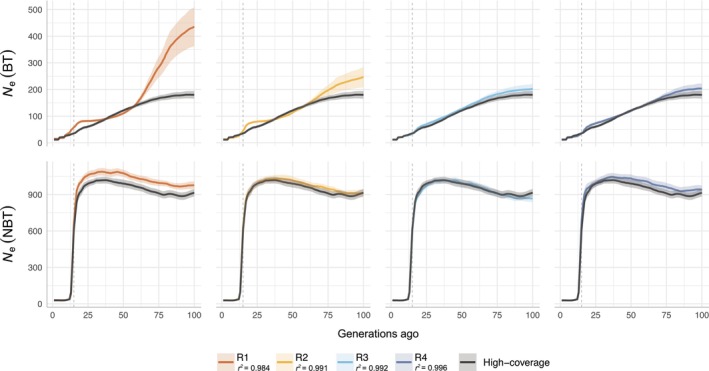
Effective population size (*N*_e_) trajectories inferred from imputed and high‐coverage datasets. *N*_e_ over the past 100 generations was inferred for the BT (top panels, *n* = 14) and NBT (bottom panels, *n* = 14) populations using GONE with the high‐coverage and imputed datasets with different imputation accuracy levels (R1–R4, from lower to higher concordance). Mean values (thick lines) and 95% confidence intervals (shaded regions) of 50 independent replicates are represented. The grey dashed vertical line marks generation 15, differentiating between recent *N*_e_ estimates, which show close agreement between imputed and high‐coverage datasets, and older *N*_e_, which exhibit increasing divergence, particularly at lowest accuracy level (R1) and in the BT population.

Demographic reconstructions inferred with GONE from imputed data converged toward those obtained from high‐coverage genomes with increasing imputation accuracy (Figure 3; Table S2). At the lowest accuracy level (R1), deviations from the high‐coverage trajectories were substantial in the BT population (Cohen's *d* = 0.62 [0.34 to 0.91]), where older effective population sizes were markedly overestimated. These discrepancies were already largely reduced at the R2 level (Cohen's *d* = 0.26 [−0.02 to 0.54]). In contrast, for the NBT population, differences between high‐coverage and imputed trajectories were less pronounced even at the lowest accuracy level (R1) (Cohen's *d* = 0.20 [−0.08 to 0.48]).

Across all imputation scenarios, *N*_e_ estimates for the most recent past (approximately the last 15 generations) were highly consistent between imputed and high‐coverage datasets, with negligible improvements as imputation accuracy increased (Figure 3; Table S2). Consequently, *N*_e_ differences were primarily confined to older generations in both populations, indicating that imputation uncertainty disproportionately affects inference of deeper demographic history rather than more recent population dynamics.

### 3.3 ROH‐Based Inbreeding Patterns Estimation

**FIGURE 4:**
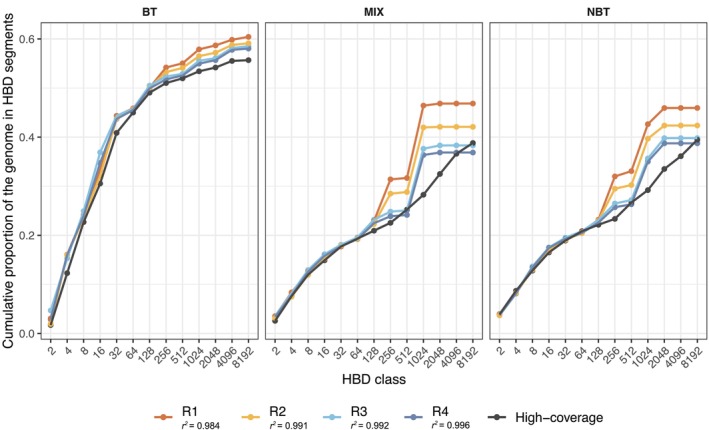
Cumulative proportion of the genome assigned to each HBD class. Mean inbreeding coefficients were estimated with RZooRoH at each HBD class for the BT, MIX, and NBT populations, from high‐coverage and imputed datasets across different imputation accuracy levels (R1–R4, from lower to higher concordance). *F*_ROH_ is generally slightly overestimated in all imputed datasets, with larger deviations at lower imputation accuracy levels and when older HBD classes are included.

*F*_ROH_ estimates obtained from imputed data were generally aligned with those derived from high‐coverage genotypes across populations and imputation accuracy levels, though a systematic slight overestimation was observed at lower imputation accuracy levels (Figure 4, Table S3). This bias was more pronounced when *F*_ROH_ was defined including higher HBD classes that include shorter ROH generated by older inbreeding events, notably from class 128 onwards. This was particularly evident in the MIX and NBT populations, where this class comprises ROH with mean lengths of 811 and 715 kb, respectively (Figure S7), reflecting inbreeding events from ~64 generations ago.

**FIGURE 5:**
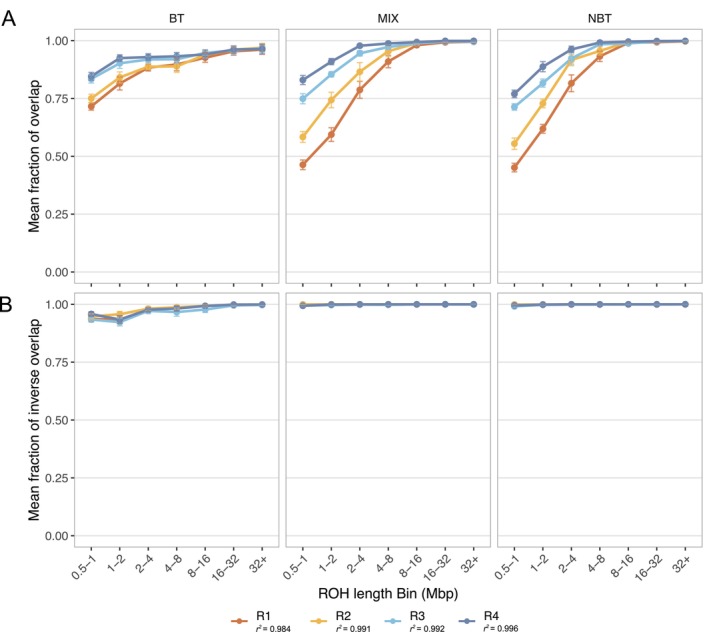
Overlap between runs of homozygosity (ROH) inferred from imputed and high‐coverage datasets. Mean per‐individual overlap fractions are shown between ROH detected in imputed datasets with different imputation accuracy levels (R1–R4, from lower to higher accuracy) and those detected in high‐coverage genotypes, across ROH length bins and populations. Error bars represent 95% confidence intervals across individuals. (A) Mean fraction of imputed ROH of a given length overlapping any high‐coverage ROH. (B) Mean fraction of high‐coverage ROH of a given length overlapping any imputed ROH. This bidirectional comparison indicates overcalling of shorter ROH in imputed datasets, particularly at lower imputation accuracy levels in the NBT and MIX populations. In contrast, most ROH identified in the high‐coverage dataset are recovered in imputed datasets.

Overlap fraction between ROH estimated from the imputed and high‐coverage datasets scaled positively with imputation accuracy and sharply increased with ROH size in MIX and NBT individuals (Figure 5A), reaching near complete overlap for ROH of 8 Mbp or more at the lowest accuracy level (R1). This pattern coincided with a consistent overcall of imputed short ROH in these populations, particularly evident in the 0.5–1 Mbp bin, and slighter up to the 2–4 Mbp bin (Figure S8). In contrast, for the BT individuals, the fraction of overlap increased with ROH size more smoothly, reaching near complete overlap only for the longest ROH sizes (Figure 5A), and did not show much improvement with higher imputation accuracy. The inverse overlap (i.e., high‐coverage ROH overlap with imputed ROH) was nearly complete across all populations and imputation accuracy levels (Figure 5B), indicating that most ROH identified in the high‐coverage dataset were also captured in the imputed datasets, regardless of their length.

Filtering out ROH with low mean HBD‐probabilities could result in the exclusion of non‐overlapping imputed ROH (false positives) (Figure S9), but it would also result in the loss of some overlapping ROH (true positives), especially at shorter ROH length bins. This establishes a direct trade‐off between false‐positive reduction and the sensitivity of true ROH signal detection across different thresholds.

### 3.4 Ancestry Characterization

**FIGURE 6:**
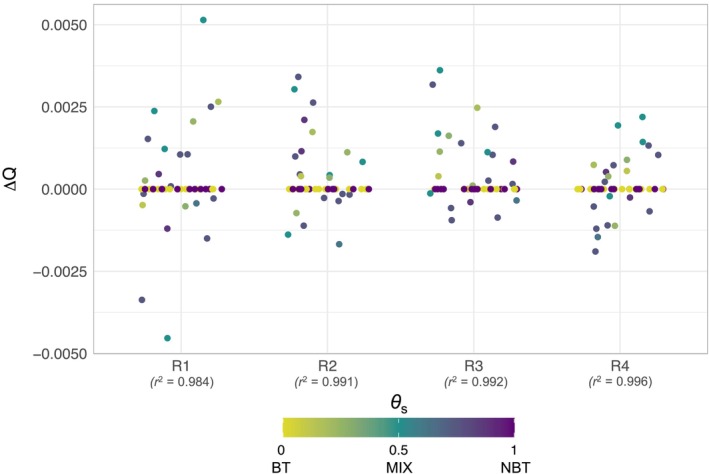
Differences in ADMIXTURE ancestry proportions between imputed and high‐coverage datasets. Per‐individual ancestry proportions (*Q* values) inferred from imputed datasets at different imputation accuracy levels (R1–R4, from lower to higher concordance) were compared to those obtained from high‐coverage genotypes. Points are coloured according to values of *θₛ*, a pedigree‐based estimate of ancestry proportions ranging from the BT population (*θₛ* = 0; green) to the NBT population (*θₛ* = 1; purple). Ancestry proportions inferred for non‐admixed individuals show minimal differences between datasets.

ADMIXTURE analyses revealed patterns of individual ancestry that were largely consistent between the high‐coverage and the imputed datasets regardless of imputation accuracy (Figure 6 and Figure S10, Table S4). In general, pure individuals from the BT and NBT populations were accurately assigned to their corresponding ancestries. In the case of MIX, discrepancies between high‐coverage and imputed data were negligible even at the lowest imputation accuracy level tested (R1) (Cohen's *d* = 0.001 (−0.002 to 0.005); Figure 6, Table S4).

**FIGURE 7:**
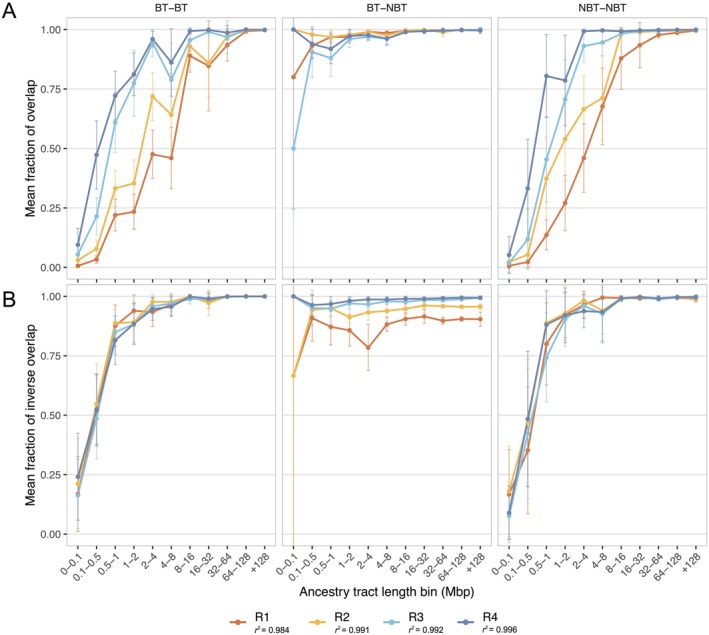
Overlap between ancestry‐specific local tracts inferred from imputed and high‐coverage datasets in admixed individuals. Mean per‐individual overlap fractions are shown between ancestry‐specific tracts identified in imputed datasets with different imputation accuracy levels (R1–R4, from lower to higher concordance) and those identified in high‐coverage genotypes, across tract length bins and populations. Error bars represent 95% confidence intervals across individuals. (A) Mean fraction of imputed tracts of a given length overlapping high‐coverage tracts of the same ancestry class. (B) Mean fraction of high‐coverage tracts of a given length overlapping imputed tracts of the same ancestry class. Shorter homozygous ancestry tracts (BT‐BT and NBT‐NBT) show reduced overlap in both directions, whereas heterozygous ancestry tracts (BT‐NBT) display relatively stable overlap across tract lengths.

Overlap between ancestry tracts inferred from the imputed and high‐coverage data depended strongly on both imputation accuracy and tract length (Figure 7). For homozygous ancestries (BT‐BT and NBT‐NBT), overlap increased markedly with tract length and imputation accuracy, displaying a high variability across individuals (Figure 7A), possibly related to their diverse admixture levels. A similar length‐dependent pattern was observed when considering inverse overlap (i.e., high‐coverage tracts overlap with imputed tracts), although overlap reached higher levels at shorter lengths (Figure 7B). In contrast, heterozygous ancestry tracts (BT‐NBT) showed generally high overlap (~95%) across tract‐length bins even at the lowest accuracy level (R1) (Figure 7A), while inverse overlap remained relatively flat at ~80% (Figure 7B). The observed overlap patterns were reflected by specific shifts in the ancestry tract length distributions across individuals within the imputed data. Specifically, imputed genotypes showed a consistent overcall of short homozygous ancestry tracts (Figure S11), particularly those shorter than 1 Mbp, and this was most pronounced with decreasing imputation accuracy. For the heterozygous ancestry, tracts longer than 32 Mbp were underrepresented in imputed data at lower accuracy levels.

Mean confusion matrices revealed that minor homozygous ancestry assignments—BT‐BT in mostly NBT individuals, and NBT‐NBT in mostly BT individuals—were more difficult to resolve with imputed data than major homozygous ancestries, without a consistent improvement with increasing imputation accuracy (Figure S12; Tables S5 and S6). This effect was particularly evident for the NBT‐NBT state in mostly BT individuals, where concordant positions between high‐coverage and imputed data ranged from 61% to 90% and showed substantial inter‐individual variability (Table S5). In contrast, BT‐BT ancestry was consistently assigned in more than 92% of positions in mostly NBT individuals in the imputed and high‐coverage datasets (Table S6). Assignments of the major homozygous ancestry states—BT‐BT in mostly BT individuals and NBT‐NBT in mostly NBT individuals—showed very high concordance in both ancestry groups (> 99%; Tables S5 and S6). For individuals with similar BT and NBT ancestry proportions (*θₛ* = 0.5), both homozygous states were generally inferred with high precision, though performance was slightly lower for NBT‐NBT tracts (Table S7). Importantly, most misclassified homozygous ancestry states were not erroneously assigned to the opposite homozygous category, but rather misclassified as heterozygous across all ancestry groups. Heterozygous ancestry inference (BT‐NBT) was also generally precise (> 92%) in all groups and showed clear imputation accuracy‐dependent improvements (Figure S12; Tables S5–S7). Moreover, misassignments of heterozygous states were approximately equally distributed between the two homozygous ancestries, indicating less directional bias than observed for homozygous ancestry classifications.

Similar to what was observed with ROH mean HBD‐probability, applying filters based on ancestry‐assignment probability could reduce the number of erroneously inferred tracts (Figure S13) with the potential trade‐off of removing correctly inferred ancestry segments. Unlike ROH filtering, where segments failing a threshold can be classified as non‐ROH, the reassignment of low‐confidence ancestry tracts depends on tract length, assigned ancestry class, and the individual's major ancestry proportion, making the post‐filter classification more variable than the binary categorization used for ROH.

## 4 Discussion

In this study, we assessed how low‐coverage whole‐genome sequencing combined with genotype imputation performs across a range of population genome‐wide and local genomic inferences in different populations of an endangered species with a complex demographic history. Using a reference panel of 50 high‐coverage Iberian lynx genomes, we evaluated imputation accuracy under different reference panel compositions, target configurations, and imputation parameters. Our results provide a structured framework to evaluate when lcWGS‐based imputation is sufficient for conservation genomics applications, when additional caution is required, and which design choices can improve imputation quality.

The extent to which genomic inferences based on imputed data are accurate depends on imputation performance. Reference panel composition emerged as a key determinant of imputation accuracy in this system, particularly given the limited number of available high‐coverage genomes. While some previous studies in humans have shown that ancestry‐matched reference panels can improve imputation accuracy even when smaller (Mitt et al. 2017; Wang et al. 2016), our results indicate that, under severe reference panel size constraints, a larger ancestry‐mixed panel (*n* = 50) can outperform ancestry‐specific alternatives (*n* ≈ 15), consistent with findings in livestock species (Rowan et al. 2019; Ye et al. 2019). This likely reflects both the reduced size of population‐specific subsets and the benefit of incorporating a broader diversity of haplotypes through admixed individuals. In this context, multi‐target imputation further improved accuracy, suggesting that sharing information across target individuals can compensate for a limited reference panel size, consistent with recent results in barn owls (Topaloudis et al. 2026).

Imputation performance can be further improved through appropriate parameter choices. In particular, we observed a substantial increase in accuracy when using a biologically realistic *N*_e_ (−ne parameter) during imputation, in line with previous studies (Jiang et al. 2022; Sun et al. 2025). The *N*_e_ definition contributes to the estimation of the recombination rate, which affects haplotype sampling and genotype probability estimation during imputation (Rubinacci et al. 2021). Default settings, originally designed for large human populations, may poorly reflect the extended linkage disequilibrium and long haplotypes present in strongly bottlenecked populations. In our case, we obtained the highest accuracy with *N*_e_ = 10, requiring considerably lower sequencing coverage compared to the default to achieve optimal performance. This parameter is therefore of special importance in endangered species with small population sizes, where maximizing imputation accuracy at minimal sequencing cost is a major objective.

In contrast, increasing the number of phasing iterations did not lead to any improvements in imputation accuracy, contrary to previous reports (Pook et al. 2020; Sun et al. 2025). This suggests that, in this system, the phasing algorithm reaches a stable solution under the recommended iteration scheme, potentially due to the extended linkage disequilibrium and small *N*_e_ characteristic of the Iberian lynx (Abascal et al. 2016). Finally, we show that filtering out low‐quality variants can further improve imputation accuracy, at the cost of increased missing data. This trade‐off may be advantageous for analyses that prioritize genotype reliability over marker density, reinforcing the idea that post‐imputation filtering can be tuned to optimize data quality in particular downstream applications.

Reduced imputation accuracy at rare variants may be particularly relevant in this context, as these variants are more prone to imputation uncertainty and post‐imputation filtering (Chat et al. 2022; Lloret‐Villas et al. 2023; Pasaniuc et al. 2012; Rubinacci et al. 2021; Teng et al. 2022; Watowich et al. 2023). Rare alleles are disproportionately represented in heterozygous genotypes, so their misimputation can generate spurious short ROH or increase their length, artificially inflating inbreeding estimates. In addition, because rare variants are often ancestry‐informative (O'Connor et al. 2015; Ros‐Freixedes et al. 2022; Tennessen et al. 2012), their filtering or inaccurate imputation may reduce the ancestry inference resolution, particularly for local ancestry tracts.

Within this framework, our results reveal a clear contrast between the robustness of genome‐wide summary statistics and the vulnerability of local genomic inferences to imputation‐related bias. Indeed, individual inbreeding was modestly overestimated when older inbreeding events were considered, and ancestry proportions displayed minor discrepancies in admixed individuals. These deviations were small and consistent with known properties of genotype imputation, including the reduced accuracy in heterozygous genotype inference from lcWGS data (Ros‐Freixedes et al. 2020; Wragg et al. 2024) or the misimputation of ancestry‐informative rare variants. Still, the robustness in these inferences suggests that genome‐wide summaries integrating information across many loci can tolerate moderate levels of genotyping error, particularly in conservation settings where relative rather than absolute estimates are often of primary interest.

In contrast, fine‐scale local genomic patterns were substantially more sensitive to imputation artefacts even under high imputation accuracy scenarios, demonstrating how few localized imputation errors can disproportionately affect downstream analyses relying on tract continuity rather than per‐site accuracy. This was particularly evident for shorter ancestry and inbreeding tracts, which reflect older demographic events as they are broken down by recombination over time. The overrepresentation of short ROH observed here is therefore likely driven by isolated imputation errors that generate spurious homozygous tracts rather than reflecting true autozygosity. Longer ROH, resulting from more recent inbred crosses (Ceballos et al. 2018; Kirin et al. 2010), can be more robust to this type of error, as homozygosity over large genomic regions is less likely to be falsely reconstructed. Similar effects have already been reported in humans (Sousa da Mota et al. 2023) and macaques (Freudiger et al. 2025), indicating that this is likely to be a general bias resulting from the use of imputed data for ROH calling. These limitations were further accentuated in the highly inbred population, where reduced heterozygosity may increase the likelihood of erroneously merging adjacent ROH, underscoring the interaction between population genomic background, demography and local inference accuracy.

Similarly, local ancestry inference in admixed individuals appeared to be particularly vulnerable to imputation errors affecting short ancestry tracts. Our results suggest that long heterozygous ancestry tracts are being fragmented by erroneously inserted short homozygous ancestry tracts when using imputed data, likely arising from misimputed homozygous genotypes interrupting otherwise continuous heterozygous ancestry regions. This interpretation is supported by the consistent overcall of short homozygous ancestry tracts, the underrepresentation of long heterozygous segments, and inverse overlap patterns indicating that long heterozygous tracts in the high‐coverage dataset are frequently interrupted in imputed datasets.

Mean confusion matrices clarified the directionality of local ancestry misassignments. Short homozygous ancestry tracts, captured by minor ancestry components, were frequently misclassified as heterozygous in the imputed data, especially in admixed individuals whose major ancestry component corresponded to the most bottlenecked population. The lack of improvement with increasing imputation accuracy and the high variability among individuals may be explained by a combination of factors: stochasticity in the downsampling of BAM files, our limited sample size (only five individuals with predominantly bottlenecked ancestry proportions), and the intrinsic rarity and short length of such homozygous ancestry tracts in admixed individuals, which makes them inherently difficult to detect. Conversely, major homozygous ancestry states were precisely inferred across imputation accuracy levels. Together, these patterns reflect the substantial impact of lcWGS and imputation on the detection of short homozygous ancestry tracts, which are generated after many generations of recombination and could be more affected by a tendency of imputation algorithms to smooth local ancestry toward the most frequent haplotypes in the reference panel (Lamb et al. 2021; Pimentel et al. 2015). These findings highlight a fundamental limitation of imputation‐based local ancestry inference when targeting ancient admixture signals captured by short tracts, particularly when using small reference panels of haplotypes.

Consistent with patterns observed for local inferences, demographic histories inferred from imputed data closely tracked those from high‐coverage genomes for more recent time periods, whereas older *N*_*e*_ estimates showed increasing bias. Because LD‐based inferences for the distant past rely on information from short physical distances (Hayes et al. 2003; Tenesa et al. 2007), imputation errors could disrupt these subtle signals more significantly than they do for the long‐range LD patterns that inform recent demographic history, highlighting the need for caution when interpreting long‐term demographic trends from imputed data, particularly at lower imputation accuracy levels.

Despite the deviations observed in the imputed data, our findings confirm that this approach can be valuable for the study of recent demographic processes, which are the most relevant in a conservation context, since they are more likely to impact current fitness (Kyriazis et al. 2025). For example, long ROH resulting from recent inbreeding are more likely to include more deleterious variants than short ROH, as purging of recessive deleterious mutations has had a limited time to act (Mathur et al. 2023; Steux and Szpiech 2024; Stoffel et al. 2021b; Szpiech et al. 2013). Likewise, the ability to recover reliable recent *N*_*e*_ trajectories remains valuable for conservation applications, as it can inform short‐term management actions and support projections of future population viability (Fedorca et al. 2024; Nadachowska‐Brzyska et al. 2022).

By enabling genome‐wide analyses in large numbers of individuals at a reduced cost, this approach also creates new opportunities to investigate the genetic basis of inbreeding depression and genetic rescue in endangered species. These could include, for example, genome‐wide association studies of fitness‐related traits (Hill et al. 2022; Stoffel et al. 2021a; Zhang et al. 2024), the characterization of genomic regions enriched or depleted in ROH (Dzomba et al. 2021; Illa et al. 2024; Mulim et al. 2022; Peripolli et al. 2018), or the analysis of changes in ancestry patterns through time following admixture (Chen et al. 2019; Gompert and Buerkle 2011).

Beyond its applications in research, the possibility of generating cost‐effective WGS data could substantially facilitate the integration of genomics into the genetic management of endangered species. More accurate estimates of realized kinship can improve strategies to support the maintenance of genetic diversity, while fine‐scale genomic information opens the door to a more direct management of fitness. For instance, the assessment of the expected ROH overlap between potential parents could prevent inbreeding more effectively, as demonstrated in Western burrowing owls (Bossu et al. 2023); and broader access to functional variation can facilitate the management of genetic disorders in captive breeding programs or contribute to reducing overall genetic load (Speak et al. 2024).

## 5 Conclusions

In this study, we provide additional practical guidance for the application of lcWGS and imputation in endangered species, including how reference‐panel composition and imputation parameters and configuration can be optimized to maximize imputation accuracy. We achieved high imputation accuracy using a small reference panel and obtained robust genome‐wide metrics relevant for conservation applications. In contrast, fine‐scale local inferences, particularly those linked to older events, were more sensitive to imputation‐related artefacts. These findings further emphasize the importance of directly benchmarking imputation performance, as similar sequencing coverages may yield different levels of accuracy depending on parameter settings and reference‐panel composition, so that sequencing coverage alone may not fully predict the reliability of downstream analyses. Overall, we show that lcWGS‐based imputation represents a cost‐effective and flexible framework for studying recent demographic processes and genome‐wide patterns in conservation genomics.

## Author Contributions

L.M.‐F. and J.A.G. designed the study. L.M.‐F., L.P.‐S. and L.S. performed laboratory work. L.M.‐F. and E.B. did the WGS data processing. L.M.‐F. and L.P.‐S. performed the population genomic analyses with advice from J.A.G. and E.B. L.M.‐F. produced the figures and wrote the paper. All authors contributed, revised and approved the final manuscript.

## Funding

This study was supported by Dirección General de Investigación Científica y Técnica (PID2021123358OBI00), Ministerio de Universidades (FPU21/02418), and Ministerio de Ciencia e Innovación (PRE2018‐083223, PRE2022‐105110).

## Conflicts of Interest

The authors declare no conflicts of interest.

## Supporting information


**Table S1:** Summary of samples with whole genome‐sequencing data included in the study. Information on the individuals' origin population, pedigree‐based ancestry coefficient (*θ*_*s*_), sex (1 for male, 2 for female), sequencing coverage and the source study from which the data were obtained.
**Table S2:** Effect sizes of the differences in demographic trajectories inferred from imputed and high‐coverage datasets. Cohen's *d* effect sizes (95% CI) comparing effective population size (*N*_e_) estimates inferred from imputed datasets at four imputation accuracy levels (R1–R4, from lower to higher concordance) against the high‐coverage dataset for the last 100 generations for the BT (*n* = 14) and NBT (*n* = 14) populations. *N*_e_ estimates were obtained with GONE, and effect sizes were computed separately for recent (1–15 generations), old (16–100 generations) and overall demographic trajectories, showing that most differences between the demographic trajectories inferred high‐coverage and the imputed datasets come from older periods of time.
**Table S3:** Effect sizes of differences in inbreeding coefficients (*F*_ROH_) between imputed and high‐coverage datasets across different HBD classes. Cohen's *d* effect sizes (95% CI) and median differences for *F*_ROH_ estimates obtained from imputed datasets at four imputation accuracy levels (R1–R4, from lower to higher concordance) relative to those derived from the high‐coverage dataset. Inbreeding coefficients were estimated across different HBD classes, reflecting inbreeding from different temporal depths. Overall, F_ROH_ estimates from imputed data tended to be inflated compared to high‐coverage data, with larger deviations observed at lower imputation accuracy levels and for older inbreeding signals (e.g., HBD class = 1024).
**Table S4:** Effect sizes of differences in ADMIXTURE ancestry proportions inferred from imputed and high‐coverage datasets. Cohen's *d* effect sizes (95% CI) and median differences comparing ADMIXTURE ancestry proportions estimated for admixed individuals (*Q*_MIX_) from imputed datasets at four imputation accuracy levels (R1–R4, from lower to higher concordance) against those obtained from the high‐coverage dataset. Differences in ancestry proportion estimates were negligible across all imputation accuracy levels.
**Table S5:** Mean confusion matrices of admixed individuals with majority of BT pedigree‐expected ancestry (0 < *θ*_*s*_ < 0.5). Cells show the proportion of SNPs assigned to each ancestry class in the imputed (columns) compared to the high‐coverage dataset (rows). R1 to R4 represent the four imputation accuracy levels tested, from lower to higher concordance. Values in parentheses indicate standard deviations across individuals. Bolded values highlight the assigned proportions matching between both datasets.
**Table S6:** Mean confusion matrices of admixed individuals with majority of NBT pedigree‐expected ancestry (0.5 < *θ*_*s*_ < 1). Proportion of SNPs assigned to each ancestry class in the imputed data across coverages (columns) compared to the high‐coverage data (rows). R1 to R4 represent the four imputation accuracy levels tested, from lower to higher concordance. Values in parentheses indicate standard deviations across individuals. Bolded values highlight the assigned proportions matching between both datasets.
**Table S7:** Mean confusion matrix of admixed individuals with the equal proportions of BT and NBT pedigree‐expected ancestry (*θ*_*s*_ = 0.5). Proportion of SNPs assigned to each ancestry class in the imputed data (columns) compared to the high‐coverage data (rows). R1 to R4 represent the four imputation accuracy levels tested, from lower to higher concordance. Values in parentheses indicate standard deviations across individuals. Bolded values highlight the assigned proportions matching between both datasets.
**Figure S1:** Distribution of pairwise kinship coefficients estimated with KING among reference panel individuals. Red vertical lines indicate the theoretical KING threshold values used to classify relatedness categories; from right to left, these correspond to duplicate/monozygotic twins (> 0.354), first‐degree relatives (0.177, 0.354), second‐degree relatives (0.0884, 0.177), and third‐degree relatives (0.0442, 0.0884).
**Figure S2:** Non‐reference discordance (NRD) rates across imputation assays. Left panel summarizes NRD rates obtained under alternative reference panel compositions—either a mixed‐reference (including all high‐coverage sequenced individuals) or a specific‐reference (including only individuals from the bottlenecked, BT, or non‐bottlenecked, NBT, populations)—and different imputation configurations, namely single‐target imputation (only the downsampled individual was imputed) and multi‐target imputation (the downsampled individual was imputed jointly with additional whole‐genome sequenced samples sharing the same ancestral background). The center panel shows NRD rates under the multi‐target, mixed‐reference imputation assay (which yielded the highest overall accuracy) across different settings of the SHAPEIT –mcmc parameter (phasing iterations). The right panel displays NRD rates for the same assay under varying effective population sizes (GLIMPSE_phase –ne parameter) during imputation. Increasing the number of phasing iterations did not lead to further improvements in imputation accuracy, whereas reducing the assumed effective population size resulted in a substantial decrease in NRD rates.
**Figure S3:** Imputation accuracy across minor allele frequency (MAF) bins and sequencing coverage levels. Accuracy was evaluated in leave‐one‐out imputation assays within a multi‐target framework using a mixed‐ancestry reference panel. It is reported as the imputation quality score (IQS; top) and *r* ^2^ (bottom), comparing results obtained with the GLIMPSE imputation –ne parameter set to its default value (left) or to 10 (right). For IQS, mean values across MAF bins are shown as thick lines, with shaded regions indicating ±1 standard deviation. The results illustrate a decline in imputation accuracy for low‐frequency variants, as well as a marked improvement when an appropriate effective population size parameter is specified.
**Figure S4:** Per‐individual genotype concordance between imputed and high‐coverage datasets. Imputation was performed within the multi‐target configuration with a mixed‐ancestry reference panel framework, under default GLIMPSE imputation –ne parameter (left) and –ne set to 10 (right). Mean genotype concordance rate (± SD) was calculated as the proportion of correctly imputed genotypes relative to high‐coverage genotypes in each sample. The solid line indicates the mean concordance across individuals and coverage levels when considering all genotypes, whereas the dashed line shows concordance calculated using only heterozygous genotypes. Squares represent mean concordance values grouped by *θ*_*s*_, a pedigree‐based ancestry coefficient ranging from pure BT ancestry (*θ*_*s*_ = 0; green) to pure NBT ancestry (*θ*_*s*_ = 1; purple). Circles denote concordance values calculated from heterozygous genotypes only. Concordance restricted to heterozygous sites is consistently lower than overall concordance, increasing with higher NBT ancestry, especially under default imputation *–*ne.
**Figure S5:** Effect of INFO filtering on imputation accuracy and marker retention when imputing with default GLIMPSE –ne parameter (A) or with –ne set to 10 (B). Left panels show the difference of imputation *r* ^2^ per minor allele frequency (MAF) bin after filtering SNPs using increasing INFO score thresholds (INFO > 0.3 to INFO > 0.9). Right panels display the proportion of SNPs removed at each coverage level under each INFO threshold.
**Figure S6:** Effect of maximum genotype probability filtering on imputation accuracy and missing data generation when imputing with GLIMPSE default –ne parameter (A) or with –ne set to 10 (B). Left panels show the difference of imputation *r* ^2^ per minor allele frequency (MAF) bin after filtering genotypes using increasing maximum genotype probability thresholds (maxGP > 0.3 to maxGP > 0.9). Right panel displays the proportion of missing genotypes introduced under each maxGP threshold.
**Figure S7:** Mean ROH length per HBD class in the high‐coverage dataset. Values are shown for HBD classes defined by RZooRoH in the BT, NBT and MIX populations, using high‐coverage genotypes. Error bars represent the 25^th^ and 75^th^ percentiles across individuals.
**Figure S8:** Number of ROH per individual across ROH length bins. Mean values are shown across ROH length bins, for each population and imputation accuracy level (R1–R4, from lower to higher concordance).
**Figure S9:** Effect of mean HBD‐probability filtering on overlapping and non‐overlapping ROH retention. Counts of overlapping and non‐overlapping ROH across length bins after applying increasing mean HBD‐probability thresholds. Bars show the number of ROH per bin and imputation accuracy level (R1–R4, from lower to higher concordance), with lighter colors indicating the total ROH and darker colors indicating ROH removed by filtering. Blue and orange bars represent non‐overlapping and overlapping ROH, respectively. Values above bars indicate the percentage of ROH removed within each category.
**Figure S10:** Ancestry proportions inferred with ADMIXTURE. Each bar represents one individual, partitioned into two ancestral components as inferred with the high‐coverage dataset (BT in light grey and NBT in darker grey). Individuals are sorted by pedigree‐based ancestry (*θ*_*s*_, black line). Colored dots represent the ancestry proportions inferred with the imputed datasets at different imputation accuracy levels (R1–R4, from lower to higher concordance).
**Figure S11:** Distribution of ancestry tract counts across tract length bins. Number of ancestry‐specific tracts per individual across tract length bins inferred with RFMix2 from high‐coverage and imputed datasets with different imputation accuracy levels (R1–R4, from lower to higher concordance). Panels show homozygous BT ancestry tracts (left), heterozygous tracts (center) and homozygous NBT ancestry tracts (right).
**Figure S12:** Accuracy of ancestry assignment in admixed individuals. Diagonal values of individual ancestry confusion matrices for admixed samples, representing the proportion of SNPs assigned to the same ancestry in both high‐coverage and imputed datasets across different imputation accuracy levels (R1–R4, from lower to higher accuracy). Each row corresponds to one individual. Tile shading reflects the proportion of SNPs correctly assigned to their ancestry category (darker colors indicate lower proportion). Individuals are ordered by their expected ancestry proportions (*θ*_*s*_), from BT (*θ*_*s*_ = 0; green) to NBT (*θ*_*s*_ = 1; purple). Uncolored tiles represent one that had no segments detected for the NBT‐NBT ancestry in the high‐coverage dataset.
**Figure S13:** Effect of ancestry‐assignment probability filtering on overlapping and non‐overlapping ancestry tracts. Counts of overlapping and non‐overlapping ancestry tracts across length bins after applying increasing mean ancestry‐assignment probability thresholds. Bars show the number of tracts per bin and imputation accuracy level (R1–R4, from lower to higher accuracy), with lighter colors indicating total tracts and darker colors indicating tracts removed by filtering. Blue and orange bars represent non‐overlapping and overlapping tracts, respectively. Values above bars indicate the percentage of tracts removed within each category.

## Data Availability

Sequences analysed in this study are available on the European Nucleotide Archive under the primary accession numbers: PRJEB12609 (Abascal et al. 2016), PRJEB44874 (Lucena‐Perez et al. 2021), and PRJEB108892 (current study). Standard code used to generate the reference panel VCF can be found at https://github.com/luciamayorf/Data_preprocessing_alignment and https://github.com/luciamayorf/Variant_calling_and_filtering. Code utilized to perform the reference panel validation and posterior downstream analyses can be found at https://github.com/luciamayorf/lcWGS_imputation_validation.

## References

[men70179-bib-0001] Abascal, F. , A. Corvelo , F. Cruz , et al. 2016. “Extreme Genomic Erosion After Recurrent Demographic Bottlenecks in the Highly Endangered Iberian Lynx.” Genome Biology 17, no. 1: 251. 10.1186/s13059-016-1090-1. 27964752 PMC5155386

[men70179-bib-0002] Alexander, D. H. , J. Novembre , and K. Lange . 2009. “Fast Model‐Based Estimation of Ancestry in Unrelated Individuals.” Genome Research 19, no. 9: 1655–1664. 10.1101/gr.094052.109. 19648217 PMC2752134

[men70179-bib-0003] Andrews, S. 2010. “*FastQC: A Quality Control Tool for High Throughput Sequencing Data*.” https://www.bioinformatics.babraham.ac.uk/projects/fastqc.

[men70179-bib-0005] Bertrand, A. R. , N. K. Kadri , L. Flori , M. Gautier , and T. Druet . 2019. “RZooRoH: An R Package to Characterize Individual Genomic Autozygosity and Identify Homozygous‐By‐Descent Segments.” Methods in Ecology and Evolution 10, no. 6: 860–866. 10.1111/2041-210X.13167.

[men70179-bib-0006] Bossu, C. M. , M. Rodriguez , C. Rayne , et al. 2023. “Genomic Approaches to Mitigating Genetic Diversity Loss in Declining Populations.” Molecular Ecology 32, no. 19: 5228–5240. 10.1111/mec.17109. 37610278

[men70179-bib-0007] Bougiouri, K. , S. G. Aninta , S. Charlton , et al. 2025. “Imputation of Ancient Canid Genomes Reveals Inbreeding History Over the Past 10,000 Years.” Proceedings of the National Academy of Sciences of the United States of America 122, no. 48: e2416980122. 10.1073/pnas.2416980122. 41284898 PMC12684900

[men70179-bib-0008] Buckley, R. M. , A. C. Harris , G.‐D. Wang , D. T. Whitaker , Y.‐P. Zhang , and E. A. Ostrander . 2022. “Best Practices for Analyzing Imputed Genotypes From Low‐Pass Sequencing in Dogs.” Mammalian Genome 33, no. 1: 213–229. 10.1007/s00335-021-09914-z. 34498136 PMC8913487

[men70179-bib-0009] Casas‐Marce, M. , E. Marmesat , L. Soriano , et al. 2017. “Spatiotemporal Dynamics of Genetic Variation in the Iberian Lynx Along Its Path to Extinction Reconstructed With Ancient DNA.” Molecular Biology and Evolution 34, no. 11: 2893–2907. 10.1093/molbev/msx222. 28962023 PMC5850336

[men70179-bib-0010] Casas‐Marce, M. , L. Soriano , J. V. López‐Bao , and J. A. Godoy . 2013. “Genetics at the Verge of Extinction: Insights From the Iberian Lynx.” Molecular Ecology 22, no. 22: 5503–5515. 10.1111/mec.12498. 24128177

[men70179-bib-0011] Ceballos, F. C. , P. K. Joshi , D. W. Clark , M. Ramsay , and J. F. Wilson . 2018. “Runs of Homozygosity: Windows Into Population History and Trait Architecture.” Nature Reviews Genetics 19, no. 4: 220–234. 10.1038/nrg.2017.109. 29335644

[men70179-bib-0013] Chat, V. , R. Ferguson , L. Morales , and T. Kirchhoff . 2022. “Ultra Low‐Coverage Whole‐Genome Sequencing as an Alternative to Genotyping Arrays in Genome‐Wide Association Studies.” Frontiers in Genetics 12: 790445. 10.3389/fgene.2021.790445. 35251117 PMC8889143

[men70179-bib-0014] Chen, N. , I. Juric , E. J. Cosgrove , et al. 2019. “Allele Frequency Dynamics in a Pedigreed Natural Population.” Proceedings of the National Academy of Sciences 116, no. 6: 2158–2164. 10.1073/pnas.1813852116. PMC6369762 30598449

[men70179-bib-0015] Chen, S. 2023. “Ultrafast One‐Pass FASTQ Data Preprocessing, Quality Control, and Deduplication Using Fastp.” iMeta 2, no. 2: e107. 10.1002/imt2.107. 38868435 PMC10989850

[men70179-bib-0016] Chen, S.‐F. , R. Dias , D. Evans , et al. 2020. “Genotype Imputation and Variability in Polygenic Risk Score Estimation.” Genome Medicine 12, no. 1: 100. 10.1186/s13073-020-00801-x. 33225976 PMC7682022

[men70179-bib-0017] Delaneau, O. , J.‐F. Zagury , M. R. Robinson , J. L. Marchini , and E. T. Dermitzakis . 2019. “Accurate, Scalable and Integrative Haplotype Estimation.” Nature Communications 10, no. 1: 5436. 10.1038/s41467-019-13225-y. PMC6882857 31780650

[men70179-bib-0018] Druet, T. , and M. Gautier . 2017. “A Model‐Based Approach to Characterize Individual Inbreeding at Both Global and Local Genomic Scales.” Molecular Ecology 26, no. 20: 5820–5841. 10.1111/mec.14324. 28815918

[men70179-bib-0019] Druet, T. , and M. Gautier . 2022. “A Hidden Markov Model to Estimate Homozygous‐By‐Descent Probabilities Associated With Nested Layers of Ancestors.” Theoretical Population Biology 145: 38–51. 10.1016/j.tpb.2022.03.001. 35283174

[men70179-bib-0020] Dzomba, E. F. , M. Chimonyo , R. Pierneef , and F. C. Muchadeyi . 2021. “Runs of Homozygosity Analysis of South African Sheep Breeds From Various Production Systems Investigated Using OvineSNP50k Data.” BMC Genomics 22, no. 1: 7. 10.1186/s12864-020-07314-2. 33407115 PMC7788743

[men70179-bib-0021] Enbody, E. D. , A. T. Sendell‐Price , C. G. Sprehn , et al. 2023. “Community‐Wide Genome Sequencing Reveals 30 Years of Darwin's Finch Evolution.” Science 381, no. 6665: eadf6218. 10.1126/science.adf6218. 37769091

[men70179-bib-0022] Ewels, P. , M. Magnusson , S. Lundin , and M. Käller . 2016. “MultiQC: Summarize Analysis Results for Multiple Tools and Samples in a Single Report.” Bioinformatics 32, no. 19: 3047–3048. 10.1093/bioinformatics/btw354. 27312411 PMC5039924

[men70179-bib-0023] Fedorca, A. , J. Mergeay , A. O. Akinyele , et al. 2024. “Dealing With the Complexity of Effective Population Size in Conservation Practice.” Evolutionary Applications 17, no. 12: e70031. 10.1111/eva.70031. 39679127 PMC11645448

[men70179-bib-0024] Figueiró, H. V. , G. Li , F. J. Trindade , et al. 2017. “Genome‐Wide Signatures of Complex Introgression and Adaptive Evolution in the Big Cats.” Science Advances 3, no. 7: e1700299. 10.1126/sciadv.1700299. 28776029 PMC5517113

[men70179-bib-0025] Flanagan, J. , X. Liu , D. Ortega‐Reyes , et al. 2024. “Population‐Specific Reference Panel Improves Imputation Quality for Genome‐Wide Association Studies Conducted on the Japanese Population.” Communications Biology 7, no. 1: 1665. 10.1038/s42003-024-07338-4. 39702642 PMC11659288

[men70179-bib-0026] Freudiger, A. , V. M. Jovanovic , Y. Huang , et al. 2025. “Estimating Realized Relatedness in Free‐Ranging Macaques by Inferring Identity‐By‐Descent Segments.” Proceedings of the National Academy of Sciences of the United States of America 122, no. 3: e2401106122. 10.1073/pnas.2401106122. 39808663 PMC11760927

[men70179-bib-0028] Fuentes‐Pardo, A. P. , and D. E. Ruzzante . 2017. “Whole‐Genome Sequencing Approaches for Conservation Biology: Advantages, Limitations and Practical Recommendations.” Molecular Ecology 26, no. 20: 5369–5406. 10.1111/mec.14264. 28746784

[men70179-bib-0029] Fuller, Z. L. , V. J. L. Mocellin , L. A. Morris , et al. 2020. “Population Genetics of the Coral *Acropora millepora* : Toward Genomic Prediction of Bleaching.” Science 369, no. 6501: eaba4674. 10.1126/science.aba4674. 32675347

[men70179-bib-0030] Goddard, M. E. , and B. J. Hayes . 2012. “Genome‐Wide Association Studies and Linkage Disequilibrium in Cattle.” In Bovine Genomics, 192–210. Wiley. 10.1002/9781118301739.ch13.

[men70179-bib-0031] Godoy, J. A. , E. Bazzicalupo , M. Casas‐Marce , et al. 2025. “Genomic Insights Into the Origin, Decline and Recovery of the Once Critically Endangered Iberian Lynx.” Molecular Ecology 34, no. 23: e17719. 10.1111/mec.17719. 40067056 PMC12684357

[men70179-bib-0032] Gompert, Z. , and C. A. Buerkle . 2011. “A Hierarchical Bayesian Model for Next‐Generation Population Genomics.” Genetics 187, no. 3: 903–917. 10.1534/genetics.110.124693. 21212231 PMC3063681

[men70179-bib-0033] Hanks, S. C. , L. Forer , S. Schönherr , et al. 2022. “Extent to Which Array Genotyping and Imputation With Large Reference Panels Approximate Deep Whole‐Genome Sequencing.” American Journal of Human Genetics 109, no. 9: 1653–1666. 10.1016/j.ajhg.2022.07.012. 35981533 PMC9502057

[men70179-bib-0035] Hayes, B. J. , P. M. Visscher , H. C. McPartlan , and M. E. Goddard . 2003. “Novel Multilocus Measure of Linkage Disequilibrium to Estimate Past Effective Population Size.” Genome Research 13, no. 4: 635–643. 10.1101/gr.387103. 12654718 PMC430161

[men70179-bib-0036] He, S. , Y. Zhao , M. F. Mette , et al. 2015. “Prospects and Limits of Marker Imputation in Quantitative Genetic Studies in European Elite Wheat ( *Triticum aestivum* L.).” BMC Genomics 16, no. 1: 168. 10.1186/s12864-015-1366-y. 25886991 PMC4364688

[men70179-bib-0037] Heidaritabar, M. , A. Huisman , K. Krivushin , et al. 2022. “Imputation to Whole‐Genome Sequence and Its Use in Genome‐Wide Association Studies for Pork Colour Traits in Crossbred and Purebred Pigs.” Frontiers in Genetics 13: 1022681. 10.3389/fgene.2022.1022681. 36303553 PMC9593086

[men70179-bib-0038] Hewett, A. M. , E. Lavanchy , A. Topaloudis , et al. 2025. “*Growth Effects and the Underlying Genetic Architecture of Inbreeding Depression in a Wild Raptor*.” p. 2025.08.17.670740. bioRxiv. 10.1101/2025.08.17.670740.

[men70179-bib-0039] Hickey, J. M. , J. Crossa , R. Babu , and G. de los Campos . 2012. “Factors Affecting the Accuracy of Genotype Imputation in Populations From Several Maize Breeding Programs.” Crop Science 52, no. 2: 654–663. 10.2135/cropsci2011.07.0358.

[men70179-bib-0040] Hill, E. W. , M. A. Stoffel , B. A. McGivney , D. E. MacHugh , and J. M. Pemberton . 2022. “Inbreeding Depression and the Probability of Racing in the Thoroughbred Horse.” Proceedings of the Royal Society B: Biological Sciences 289, no. 1977: 20220487. 10.1098/rspb.2022.0487. PMC9240673 35765835

[men70179-bib-0041] Illa, S. K. , S. Mumtaz , S. Nath , S. Mukherjee , and A. Mukherjee . 2024. “Characterization of Runs of Homozygosity Revealed Genomic Inbreeding and Patterns of Selection in Indigenous Sahiwal Cattle.” Journal of Applied Genetics 65, no. 1: 167–180. 10.1007/s13353-023-00816-1. 38110827

[men70179-bib-0042] Jiang, Y. , H. Song , H. Gao , Q. Zhang , and X. Ding . 2022. “Exploring the Optimal Strategy of Imputation From SNP Array to Whole‐Genome Sequencing Data in Farm Animals.” Frontiers in Genetics 13: 963654. 10.3389/fgene.2022.963654. 36092888 PMC9459117

[men70179-bib-0043] Kaelin, C. B. , K. A. McGowan , A. D. Hutcherson , et al. 2024. “Ancestry Dynamics and Trait Selection in a Designer Cat Breed.” Current Biology 34, no. 7: 1506–1518.e7. 10.1016/j.cub.2024.02.075. 38531359 PMC11162505

[men70179-bib-0044] Kirin, M. , R. McQuillan , C. S. Franklin , H. Campbell , P. M. McKeigue , and J. F. Wilson . 2010. “Genomic Runs of Homozygosity Record Population History and Consanguinity.” PLoS One 5, no. 11: e13996. 10.1371/journal.pone.0013996. 21085596 PMC2981575

[men70179-bib-0045] Kleinman‐Ruiz, D. , M. Lucena‐Perez , B. Villanueva , et al. 2022. “Purging of Deleterious Burden in the Endangered Iberian Lynx.” Proceedings of the National Academy of Sciences of the United States of America 119, no. 11: e2110614119. 10.1073/pnas.2110614119. 35238662 PMC8931242

[men70179-bib-0046] Kleinman‐Ruiz, D. , B. Martínez‐Cruz , L. Soriano , et al. 2017. “Novel Efficient Genome‐Wide SNP Panels for the Conservation of the Highly Endangered Iberian Lynx.” BMC Genomics 18, no. 1: 556. 10.1186/s12864-017-3946-5. 28732460 PMC5522595

[men70179-bib-0047] Kleinman‐Ruiz, D. , L. Soriano , M. Casas‐Marce , et al. 2019. “Genetic Evaluation of the Iberian Lynx Ex Situ Conservation Programme.” Heredity 123, no. 5: 5. 10.1038/s41437-019-0217-z. PMC6972747 30952964

[men70179-bib-0048] Kyriazis, C. C. , J. A. Robinson , and K. E. Lohmueller . 2025. “Long Runs of Homozygosity Are Reliable Genomic Markers of Inbreeding Depression.” Trends in Ecology & Evolution 40: 874–884. 10.1016/j.tree.2025.06.013. 40752972 PMC13238715

[men70179-bib-0049] Lamb, H. J. , B. J. Hayes , I. A. S. Randhawa , L. T. Nguyen , and E. M. Ross . 2021. “Genomic Prediction Using Low‐Coverage Portable Nanopore Sequencing.” PLoS One 16, no. 12: e0261274. 10.1371/journal.pone.0261274. 34910782 PMC8673642

[men70179-bib-0050] Lan, T. , H. Li , B. Liu , et al. 2025. “Revealing Extensive Inbreeding and Less Efficient Purging of Deleterious Mutations in Wild Amur Tigers in China.” Journal of Genetics and Genomics 52, no. 5: 641–649. 10.1016/j.jgg.2024.12.004. 39674273

[men70179-bib-0051] Larison, B. , G. M. Pinho , A. Haghani , et al. 2021. “Epigenetic Models Developed for Plains Zebras Predict Age in Domestic Horses and Endangered Equids.” Communications Biology 4, no. 1: 1–9. 10.1038/s42003-021-02935-z. 34921240 PMC8683477

[men70179-bib-0052] Lau, W. , A. Ali , H. Maude , T. Andrew , D. M. Swallow , and N. Maniatis . 2024. “The Hazards of Genotype Imputation When Mapping Disease Susceptibility Variants.” Genome Biology 25, no. 1: 7. 10.1186/s13059-023-03140-3. 38172955 PMC10763476

[men70179-bib-0053] Li, G. , H. V. Figueiró , E. Eizirik , and W. J. Murphy . 2019. “Recombination‐Aware Phylogenomics Reveals the Structured Genomic Landscape of Hybridizing Cat Species.” Molecular Biology and Evolution 36, no. 10: 2111–2126. 10.1093/molbev/msz139. 31198971 PMC6759079

[men70179-bib-0054] Li, G. , L. W. Hillier , R. A. Grahn , et al. 2016. “A High‐Resolution SNP Array‐Based Linkage Map Anchors a New Domestic Cat Draft Genome Assembly and Provides Detailed Patterns of Recombination.” G3: Genes, Genomes, Genetics 6, no. 6: 1607–1616. 10.1534/g3.116.028746. 27172201 PMC4889657

[men70179-bib-0055] Li, H. 2013. “*Aligning Sequence Reads, Clone Sequences and Assembly Contigs With BWA‐MEM* (arXiv:1303.3997).” arXiv. 10.48550/arXiv.1303.3997.

[men70179-bib-0056] Li, H. , B. Handsaker , A. Wysoker , et al. 2009. “The Sequence Alignment/Map Format and SAMtools.” Bioinformatics 25, no. 16: 2078–2079. 10.1093/bioinformatics/btp352. 19505943 PMC2723002

[men70179-bib-0057] Li, J. , C. A. Mazur , T. Berisa , and J. K. Pickrell . 2021. “Low‐Pass Sequencing Increases the Power of GWAS and Decreases Measurement Error of Polygenic Risk Scores Compared to Genotyping Arrays.” Genome Research 31, no. 4: 529–537. 10.1101/gr.266486.120. 33536225 PMC8015847

[men70179-bib-0058] Lin, P. , S. M. Hartz , Z. Zhang , et al. 2010. “A New Statistic to Evaluate Imputation Reliability.” PLoS One 5, no. 3: e9697. 10.1371/journal.pone.0009697. 20300623 PMC2837741

[men70179-bib-0059] Liu, S. , K. E. Martin , W. M. Snelling , et al. 2024. “Accurate Genotype Imputation From Low‐Coverage Whole‐Genome Sequencing Data of Rainbow Trout.” G3: Genes, Genomes, Genetics 14, no. 9: jkae168. 10.1093/g3journal/jkae168. 39041837 PMC11373650

[men70179-bib-0060] Lloret‐Villas, A. , H. Pausch , and A. S. Leonard . 2023. “The Size and Composition of Haplotype Reference Panels Impact the Accuracy of Imputation From Low‐Pass Sequencing in Cattle.” Genetics Selection Evolution 55, no. 1: 33. 10.1186/s12711-023-00809-y. PMC10173671 37170101

[men70179-bib-0061] Lou, R. N. , A. Jacobs , A. P. Wilder , and N. O. Therkildsen . 2021. “A Beginner's Guide to Low‐Coverage Whole Genome Sequencing for Population Genomics.” Molecular Ecology 30, no. 23: 5966–5993. 10.1111/mec.16077. 34250668

[men70179-bib-0062] Lucena‐Perez, M. , D. Kleinman‐Ruiz , E. Marmesat , A. P. Saveljev , K. Schmidt , and J. A. Godoy . 2021. “Bottleneck‐Associated Changes in the Genomic Landscape of Genetic Diversity in Wild Lynx Populations.” Evolutionary Applications 14, no. 11: 2664–2679. 10.1111/eva.13302. 34815746 PMC8591332

[men70179-bib-0063] Lucena‐Perez, M. , J. L. A. Paijmans , F. Nocete , et al. 2024. “Recent Increase in Species‐Wide Diversity After Interspecies Introgression in the Highly Endangered Iberian Lynx.” Nature Ecology & Evolution 8, no. 2: 282–292. 10.1038/s41559-023-02267-7. 38225424

[men70179-bib-0064] Manichaikul, A. , J. C. Mychaleckyj , S. S. Rich , K. Daly , M. Sale , and W.‐M. Chen . 2010. “Robust Relationship Inference in Genome‐Wide Association Studies.” Bioinformatics 26, no. 22: 2867–2873. 10.1093/bioinformatics/btq559. 20926424 PMC3025716

[men70179-bib-0065] Maples, B. K. , S. Gravel , E. E. Kenny , and C. D. Bustamante . 2013. “RFMix: A Discriminative Modeling Approach for Rapid and Robust Local‐Ancestry Inference.” American Journal of Human Genetics 93, no. 2: 278–288. 10.1016/j.ajhg.2013.06.020. 23910464 PMC3738819

[men70179-bib-0066] Marchini, J. , and B. Howie . 2010. “Genotype Imputation for Genome‐Wide Association Studies.” Nature Reviews Genetics 11, no. 7: 499–511. 10.1038/nrg2796. 20517342

[men70179-bib-0067] Martin, M. , M. Patterson , S. Garg , et al. 2016. “*WhatsHap: Fast and Accurate Read‐Based Phasing*.” p. 085050. bioRxiv. 10.1101/085050.

[men70179-bib-0068] Mathur, S. , J. M. Tomeček , L. A. Tarango‐Arámbula , R. M. Perez , and J. A. DeWoody . 2023. “An Evolutionary Perspective on Genetic Load in Small, Isolated Populations as Informed by Whole Genome Resequencing and Forward‐Time Simulations.” Evolution 77, no. 3: 690–704. 10.1093/evolut/qpac061. 36626799

[men70179-bib-0069] McKenna, A. , M. Hanna , E. Banks , et al. 2010. “The Genome Analysis Toolkit: A MapReduce Framework for Analyzing Next‐Generation DNA Sequencing Data.” Genome Research 20, no. 9: 1297–1303. 10.1101/gr.107524.110. 20644199 PMC2928508

[men70179-bib-0070] McLaughlin, J. F. , and K. Winker . 2020. “An Empirical Examination of Sample Size Effects on Population Demographic Estimates in Birds Using Single Nucleotide Polymorphism (SNP) Data.” PeerJ 8: e9939. 10.7717/peerj.9939. 32995092 PMC7501783

[men70179-bib-0071] Mitt, M. , M. Kals , K. Pärn , et al. 2017. “Improved Imputation Accuracy of Rare and Low‐Frequency Variants Using Population‐Specific High‐Coverage WGS‐Based Imputation Reference Panel.” European Journal of Human Genetics 25, no. 7: 869–876. 10.1038/ejhg.2017.51. 28401899 PMC5520064

[men70179-bib-0072] Mulim, H. A. , L. F. Brito , L. F. B. Pinto , et al. 2022. “Characterization of Runs of Homozygosity, Heterozygosity‐Enriched Regions, and Population Structure in Cattle Populations Selected for Different Breeding Goals.” BMC Genomics 23, no. 1: 209. 10.1186/s12864-022-08384-0. 35291953 PMC8925140

[men70179-bib-0073] Nadachowska‐Brzyska, K. , M. Konczal , and W. Babik . 2022. “Navigating the Temporal Continuum of Effective Population Size.” Methods in Ecology and Evolution 13, no. 1: 22–41. 10.1111/2041-210X.13740.

[men70179-bib-0074] Nguyen, T. V. , S. Bolormaa , C. M. Reich , et al. 2024. “Empirical Versus Estimated Accuracy of Imputation: Optimising Filtering Thresholds for Sequence Imputation.” Genetics Selection Evolution 56, no. 1: 72. 10.1186/s12711-024-00942-2. PMC11566673 39548370

[men70179-bib-0075] Ni, G. , T. M. Strom , H. Pausch , et al. 2015. “Comparison Among Three Variant Callers and Assessment of the Accuracy of Imputation From SNP Array Data to Whole‐Genome Sequence Level in Chicken.” BMC Genomics 16, no. 1: 824. 10.1186/s12864-015-2059-2. 26486989 PMC4618161

[men70179-bib-0076] O'Connor, T. D. , W. Fu , NHLBI GO Exome Sequencing Project , et al. 2015. “Rare Variation Facilitates Inferences of Fine‐Scale Population Structure in Humans.” Molecular Biology and Evolution 32, no. 3: 653–660. 10.1093/molbev/msu326. 25415970 PMC4327153

[men70179-bib-0077] Okonechnikov, K. , A. Conesa , and F. García‐Alcalde . 2016. “Qualimap 2: Advanced Multi‐Sample Quality Control for High‐Throughput Sequencing Data.” Bioinformatics 32, no. 2: 292–294. 10.1093/bioinformatics/btv566. 26428292 PMC4708105

[men70179-bib-0078] Pasaniuc, B. , N. Rohland , P. J. McLaren , et al. 2012. “Extremely Low‐Coverage Sequencing and Imputation Increases Power for Genome‐Wide Association Studies.” Nature Genetics 44, no. 6: 631–635. 10.1038/ng.2283. 22610117 PMC3400344

[men70179-bib-0079] Pedersen, B. S. , and A. R. Quinlan . 2018. “Mosdepth: Quick Coverage Calculation for Genomes and Exomes.” Bioinformatics 34, no. 5: 867–868. 10.1093/bioinformatics/btx699. 29096012 PMC6030888

[men70179-bib-0080] Peripolli, E. , N. B. Stafuzza , D. P. Munari , et al. 2018. “Assessment of Runs of Homozygosity Islands and Estimates of Genomic Inbreeding in Gyr ( *Bos indicus* ) Dairy Cattle.” BMC Genomics 19, no. 1: 34. 10.1186/s12864-017-4365-3. 29316879 PMC5759835

[men70179-bib-0081] Pimentel, E. C. G. , C. Edel , R. Emmerling , and K.‐U. Götz . 2015. “How Imputation Errors Bias Genomic Predictions.” Journal of Dairy Science 98, no. 6: 4131–4138. 10.3168/jds.2014-9170. 25841966

[men70179-bib-0082] Pook, T. , M. Mayer , J. Geibel , et al. 2020. “Improving Imputation Quality in BEAGLE for Crop and Livestock Data.” G3: Genes, Genomes, Genetics 10, no. 1: 177–188. 10.1534/g3.119.400798. 31676508 PMC6945036

[men70179-bib-0083] Poplin, R. , P.‐C. Chang , D. Alexander , et al. 2018. “A Universal SNP and Small‐Indel Variant Caller Using Deep Neural Networks.” Nature Biotechnology 36, no. 10: 10. 10.1038/nbt.4235. 30247488

[men70179-bib-0084] Puckett, E. E. , I. S. Davis , D. C. Harper , et al. 2023. “Genetic Architecture and Evolution of Color Variation in American Black Bears.” Current Biology 33, no. 1: 86–97.e10. 10.1016/j.cub.2022.11.042. 36528024 PMC10039708

[men70179-bib-0085] Purcell, S. , B. Neale , K. Todd‐Brown , et al. 2007. “PLINK: A Tool Set for Whole‐Genome Association and Population‐Based Linkage Analyses.” American Journal of Human Genetics 81, no. 3: 559–575. 10.1086/519795. 17701901 PMC1950838

[men70179-bib-0086] Quinlan, A. R. , and I. M. Hall . 2010. “BEDTools: A Flexible Suite of Utilities for Comparing Genomic Features.” Bioinformatics 26, no. 6: 841–842. 10.1093/bioinformatics/btq033. 20110278 PMC2832824

[men70179-bib-0087] Ros‐Freixedes, R. , B. D. Valente , C.‐Y. Chen , et al. 2022. “Rare and Population‐Specific Functional Variation Across Pig Lines.” Genetics, Selection, Evolution: GSE 54: 39. 10.1186/s12711-022-00732-8. 35659233 PMC9164375

[men70179-bib-0088] Ros‐Freixedes, R. , A. Whalen , G. Gorjanc , A. J. Mileham , and J. M. Hickey . 2020. “Evaluation of Sequencing Strategies for Whole‐Genome Imputation With Hybrid Peeling.” Genetics Selection Evolution 52, no. 1: 18. 10.1186/s12711-020-00537-7. PMC7132986 32248818

[men70179-bib-0089] Rowan, T. N. , J. L. Hoff , T. E. Crum , J. F. Taylor , R. D. Schnabel , and J. E. Decker . 2019. “A Multi‐Breed Reference Panel and Additional Rare Variants Maximize Imputation Accuracy in Cattle.” Genetics, Selection, Evolution: GSE 51: 77. 10.1186/s12711-019-0519-x. 31878893 PMC6933688

[men70179-bib-0090] Rubinacci, S. , D. M. Ribeiro , R. J. Hofmeister , and O. Delaneau . 2021. “Efficient Phasing and Imputation of Low‐Coverage Sequencing Data Using Large Reference Panels.” Nature Genetics 53, no. 1: 120–126. 10.1038/s41588-020-00756-0. 33414550

[men70179-bib-0091] Santiago, E. , I. Novo , A. F. Pardiñas , M. Saura , J. Wang , and A. Caballero . 2020. “Recent Demographic History Inferred by High‐Resolution Analysis of Linkage Disequilibrium.” Molecular Biology and Evolution 37, no. 12: 3642–3653. 10.1093/molbev/msaa169. 32642779

[men70179-bib-0092] Slatkin, M. 2008. “Linkage Disequilibrium—Understanding the Evolutionary Past and Mapping the Medical Future.” Nature Reviews Genetics 9, no. 6: 477–485. 10.1038/nrg2361. PMC5124487 18427557

[men70179-bib-0093] Sousa da Mota, B. , S. Rubinacci , D. I. Cruz Dávalos , et al. 2023. “Imputation of Ancient Human Genomes.” Nature Communications 14, no. 1: 3660. 10.1038/s41467-023-39202-0. PMC10282092 37339987

[men70179-bib-0094] Speak, S. A. , T. Birley , C. Bortoluzzi , et al. 2024. “Genomics‐Informed Captive Breeding Can Reduce Inbreeding Depression and the Genetic Load in Zoo Populations.” Molecular Ecology Resources 24, no. 7: e13967. 10.1111/1755-0998.13967. 38727721

[men70179-bib-0095] Steux, C. , and Z. A. Szpiech . 2024. “*The Maintenance of Deleterious Variation in Wild Chinese Rhesus Macaques*.” p. 2023.10.04.560901. bioRxiv. 10.1101/2023.10.04.560901. PMC11157460 38795368

[men70179-bib-0096] Stoffel, M. A. , S. E. Johnston , J. G. Pilkington , and J. M. Pemberton . 2021a. “Genetic Architecture and Lifetime Dynamics of Inbreeding Depression in a Wild Mammal.” Nature Communications 12, no. 1: 2972. 10.1038/s41467-021-23222-9. PMC8138023 34016997

[men70179-bib-0097] Stoffel, M. A. , S. E. Johnston , J. G. Pilkington , and J. M. Pemberton . 2021b. “Mutation Load Decreases With Haplotype Age in Wild Soay Sheep.” Evolution Letters 5, no. 3: 187–195. 10.1002/evl3.229. 34136268 PMC8190445

[men70179-bib-0098] Sun, H. , X. Wang , W. Cai , et al. 2025. “Optimizing Genotype Imputation Strategy for Low‐Coverage Whole‐Genome Resequencing and Investigating Genomic Selection Signatures in Domesticated *Paralichthys olivaceus* .” Aquaculture Reports 42: 102826. 10.1016/j.aqrep.2025.102826.

[men70179-bib-0099] Supple, M. A. , and B. Shapiro . 2018. “Conservation of Biodiversity in the Genomics Era.” Genome Biology 19, no. 1: 131. 10.1186/s13059-018-1520-3. 30205843 PMC6131752

[men70179-bib-0100] Szpiech, Z. A. , J. Xu , T. J. Pemberton , et al. 2013. “Long Runs of Homozygosity Are Enriched for Deleterious Variation.” American Journal of Human Genetics 93, no. 1: 90–102. 10.1016/j.ajhg.2013.05.003. 23746547 PMC3710769

[men70179-bib-0101] Tan, H. Z. , K. C. Stuart , T. Vi , et al. 2025. “High Imputation Accuracy Can be Achieved Using a Small Reference Panel in a Natural Population With Low Genetic Diversity.” Molecular Ecology Resources 25: e70024. 10.1111/1755-0998.70024. 40797301 PMC12550488

[men70179-bib-0102] Tenesa, A. , P. Navarro , B. J. Hayes , et al. 2007. “Recent Human Effective Population Size Estimated From Linkage Disequilibrium.” Genome Research 17, no. 4: 520–526. 10.1101/gr.6023607. 17351134 PMC1832099

[men70179-bib-0103] Teng, J. , C. Zhao , D. Wang , et al. 2022. “Assessment of the Performance of Different Imputation Methods for Low‐Coverage Sequencing in Holstein Cattle.” Journal of Dairy Science 105, no. 4: 3355–3366. 10.3168/jds.2021-21360. 35151474

[men70179-bib-0104] Tennessen, J. A. , A. W. Bigham , T. D. O'Connor , et al. 2012. “Evolution and Functional Impact of Rare Coding Variation From Deep Sequencing of Human Exomes.” Science 337, no. 6090: 64–69. 10.1126/science.1219240. 22604720 PMC3708544

[men70179-bib-0105] Topaloudis, A. , T. Cumer , E. Lavanchy , et al. 2026. “Benchmarking Imputation Accuracy in the Presence or Absence of a Reference Panel.” Molecular Biology and Evolution 43, no. 4: msag094. 10.1093/molbev/msag094. 41967458 PMC13122032

[men70179-bib-0106] Torchiano, M. 2016. “*Effsize—A Package for Efficient Effect Size Computation* [Computer Software].” Zenodo. 10.5281/zenodo.196082.

[men70179-bib-0108] Vi, T. , K. C. Stuart , H. Z. Tan , A. Lloret‐Villas , and A. W. Santure . 2025. “Assessing Genotype Imputation Methods for Low‐Coverage Sequencing Data in Populations With Differing Relatedness and Inbreeding Levels.” Molecular Ecology Resources 25: e70049. 10.1111/1755-0998.70049. 41017706 PMC12550480

[men70179-bib-0109] Wang, X. , C.‐Y. Cheng , J. Liao , et al. 2016. “Evaluation of Transethnic Fine Mapping With Population‐Specific and Cosmopolitan Imputation Reference Panels in Diverse Asian Populations.” European Journal of Human Genetics 24, no. 4: 592–599. 10.1038/ejhg.2015.150. 26130488 PMC4929869

[men70179-bib-0110] Watowich, M. M. , K. L. Chiou , B. Graves , et al. 2023. “Best Practices for Genotype Imputation From Low‐Coverage Sequencing Data in Natural Populations.” Molecular Ecology Resources 25: e13854. 10.1111/1755-0998.13854. 37602981 PMC10879460

[men70179-bib-0111] Weng, Z.‐Q. , M. Saatchi , R. D. Schnabel , J. F. Taylor , and D. J. Garrick . 2014. “Recombination Locations and Rates in Beef Cattle Assessed From Parent‐Offspring Pairs.” Genetics Selection Evolution 46, no. 1: 34. 10.1186/1297-9686-46-34. PMC4071795 24885305

[men70179-bib-0112] Westbury, M. V. , R. Barnett , M. Sandoval‐Velasco , et al. 2021. “A Genomic Exploration of the Early Evolution of Extant Cats and Their Sabre‐Toothed Relatives.” Open Research Europe 1: 25. 10.12688/openreseurope.13104.2. 35098251 PMC7612286

[men70179-bib-0113] Wickham, H. 2016. Ggplot2. Springer International Publishing. 10.1007/978-3-319-24277-4.

[men70179-bib-0114] Wragg, D. , W. Zhang , S. Peterson , et al. 2024. “A Cautionary Tale of Low‐Pass Sequencing and Imputation With Respect to Haplotype Accuracy.” Genetics Selection Evolution 56, no. 1: 6. 10.1186/s12711-024-00875-w. PMC10785484 38216889

[men70179-bib-0115] Wu, S.‐X. , Q.‐F. Zeng , W.‐T. Han , et al. 2024. “Deciphering the Population Structure and Genetic Basis of Growth Traits From Whole‐Genome Resequencing of the Leopard Coral Grouper ( *Plectropomus leopardus* ).” Zoological Research 45, no. 2: 329–340. 10.24272/j.issn.2095-8137.2023.270. 38485503 PMC11017084

[men70179-bib-0116] Yang, B. , Y. Li , Q. Li , and S. Liu . 2024. “High‐Throughput and Cost‐Effective Genotyping by Low‐Coverage Whole Genome Sequencing With Genotype Imputation in Pacific Oyster, *Crassostrea gigas* .” Aquaculture 591: 741134. 10.1016/j.aquaculture.2024.741134.

[men70179-bib-0117] Ye, S. , N. Gao , R. Zheng , et al. 2019. “Strategies for Obtaining and Pruning Imputed Whole‐Genome Sequence Data for Genomic Prediction.” Frontiers in Genetics 10: 673. 10.3389/fgene.2019.00673. 31379929 PMC6650575

[men70179-bib-0118] Yun, T. , H. Li , P.‐C. Chang , M. F. Lin , A. Carroll , and C. Y. McLean . 2021. “Accurate, Scalable Cohort Variant Calls Using DeepVariant and GLnexus.” Bioinformatics 36, no. 24: 5582–5589. 10.1093/bioinformatics/btaa1081. 33399819 PMC8023681

[men70179-bib-0119] Zavattari, P. , E. Deidda , M. Whalen , et al. 2000. “Major Factors Influencing Linkage Disequilibrium by Analysis of Different Chromosome Regions in Distinct Populations: Demography, Chromosome Recombination Frequency and Selection.” Human Molecular Genetics 9, no. 20: 2947–2957. 10.1093/hmg/9.20.2947. 11115838

[men70179-bib-0120] Zhang, Z. , W. Zhao , Z. Wang , Y. Pan , Q. Wang , and Z. Zhang . 2024. “Integration of ssGWAS and ROH Analyses for Uncovering Genetic Variants Associated With Reproduction Traits in Large White Pigs.” Animal Genetics 55, no. 5: 714–724. 10.1111/age.13465. 39129705

