# Supplementary material for: Imputation in the Wild: Genome‐Wide Robustness and Fine‐Scale Limitations of Low‐Coverage Genomes in Endangered Species
Source: Mol Ecol Resour. 2026 Jul 27;26(5):e70179. doi: 10.1111/1755-0998.70179 (PMC13402980; doi:10.1111/1755-0998.70179)
Supplement: Supplementary file 1 — Table S1: Summary of samples with whole genome‐sequencing data included in the study. Information on the individuals' origin population, pedigree‐based ancestry coefficient (θ s ), sex (1 for male, 2 for female), sequencing coverage and the source study from which the data were obtained. Table S2: Effect sizes of the differences in demographic trajectories inferred from imputed and high‐coverage datasets. Cohen's d effect sizes (95% CI) comparing effective population size (N e) estimates inferred from imputed datasets at four imputation accuracy levels (R1–R4, from lower to higher concordance) against the high‐coverage dataset for the last 100 generations for the BT (n = 14) and NBT (n = 14) populations. N e estimates were obtained with GONE, and effect sizes were computed separately for recent (1–15 generations), old (16–100 generations) and overall demographic trajectories, showing that most differences between the demographic trajectories inferred high‐coverage and the imputed datasets come from older periods of time. Table S3: Effect sizes of differences in inbreeding coefficients (F ROH) between imputed and high‐coverage datasets across different HBD classes. Cohen's d effect sizes (95% CI) and median differences for F ROH estimates obtained from imputed datasets at four imputation accuracy levels (R1–R4, from lower to higher concordance) relative to those derived from the high‐coverage dataset. Inbreeding coefficients were estimated across different HBD classes, reflecting inbreeding from different temporal depths. Overall, FROH estimates from imputed data tended to be inflated compared to high‐coverage data, with larger deviations observed at lower imputation accuracy levels and for older inbreeding signals (e.g., HBD class = 1024). Table S4: Effect sizes of differences in ADMIXTURE ancestry proportions inferred from imputed and high‐coverage datasets. Cohen's d effect sizes (95% CI) and median differences comparing ADMIXTURE ancestry proportions estimated fo [file MEN-26-e70179-s001.pdf]

Title

Imputation in the wild: genome-wide robustness and fine-scale limitations of low-coverage genomes in endangered species

Running title: Evaluation of imputed genome-wide data

Authors

Lucía Mayor-Fidalgo[1], Enrico Bazzicalupo[1], Laia Pérez-Sorribes[1], Laura Soriano[1], José A. Godoy[1*]

[1] Departamento de Ecología y Evolución, Estación Biológica de Doñana, Consejo Superior de Investigaciones Científicas, Sevilla, 41092, Spain

SUPPLEMENTARY TABLES

Table S1. Summary of samples with whole genome-sequencing data included in the study. Information on the individuals' origin population, pedigree-based ancestry coefficient (θ_s), sex (1 for male, 2 for female), sequencing coverage and the source study from which the data were obtained.

Sample code	Population	θ_s	Sex	Coverage	Study
c_lp_ca_0692	NBT	1	2	39.59	This study (reference panel)
c_lp_ca_0693	MIX	0.75	1	41.18	This study (reference panel)
c_lp_ca_0756	MIX	0.5	2	33.70	This study (reference panel)
c_lp_ca_0765	MIX	0.75	2	38.23	This study (reference panel)
c_lp_ca_0800	MIX	0.75	2	31.14	This study (reference panel)
c_lp_ca_0805	NBT	1	1	50.65	This study (reference panel)
c_lp_ca_0829	MIX	0.875	1	32.28	This study (reference panel)
c_lp_ca_0855	MIX	0.875	1	39.59	This study (reference panel)
c_lp_ca_1363	MIX	0.75	1	41.85	This study (reference panel)
c_lp_ca_1372	NBT	1	1	37.22	This study (reference panel)
c_lp_ca_1430	MIX	0.625	1	31.98	This study (reference panel)
c_lp_ca_1431	MIX	0.75	2	35.55	This study (reference panel)
c_lp_ca_1480	NBT	1	1	40.89	This study (reference panel)
c_lp_ca_1812	MIX	0.75	2	39.54	This study (reference panel)
c_lp_ca_1814	MIX	0.75	1	42.76	This study (reference panel)
c_lp_ca_1819	MIX	0.75	2	37.02	This study (reference panel)
c_lp_do_0001	BT	0	2	29.80	This study (reference panel)
c_lp_do_0005	BT	0	1	42.86	This study (reference panel)
c_lp_do_0010	BT	0	2	40.86	This study (reference panel)
c_lp_do_0202	BT	0	2	32.68	This study (reference panel)
c_lp_do_0205	BT	0	2	35.87	This study (reference panel)
c_lp_do_0215	BT	0	2	34.82	This study (reference panel)
c_lp_do_0219	BT	0	2	43.99	This study (reference panel)
c_lp_do_0256	BT	0	2	44.06	This study (reference panel)
c_lp_do_0260	BT	0	1	48.30	This study (reference panel)
c_lp_do_0290	MIX	0.5	1	33.71	This study (reference panel)
c_lp_do_0380	MIX	0.5	1	33.01	This study (reference panel)
c_lp_do_0384	BT	0	2	41.17	This study (reference panel)
c_lp_do_0385	BT	0	2	60.64	This study (reference panel)
c_lp_do_0451	BT	0	1	35.92	This study (reference panel)
c_lp_do_0601	MIX	0.25	1	37.05	This study (reference panel)
c_lp_do_0611	BT	0	2	36.38	This study (reference panel)
c_lp_do_0683	MIX	0.5	1	36.90	This study (reference panel)
c_lp_do_0773	BT	0	2	36.62	This study (reference panel)
c_lp_do_0784	MIX	0.125	1	39.59	This study (reference panel)
c_lp_do_1607	MIX	0.1875	1	30.19	This study (reference panel)
c_lp_do_1849	MIX	0.75	2	39.79	This study (reference panel)
c_lp_do_1858	MIX	0.25	1	41.75	This study (reference panel)
c_lp_do_1860	BT	0	2	38.22	This study (reference panel)
c_lp_do_2046	MIX	0.2813	1	34.85	This study (reference panel)
c_lp_sm_0211	NBT	1	2	33.58	Bazzicalupo et al., 2026 (reference panel)
c_lp_sm_0220	NBT	1	1	43.32	Bazzicalupo et al., 2026 (reference panel)
c_lp_sm_0225	NBT	1	2	33.88	Bazzicalupo et al., 2026 (reference panel)
c_lp_sm_0239	NBT	1	1	40.94	Bazzicalupo et al., 2026 (reference panel)
c_lp_sm_0278	NBT	1	2	39.22	Bazzicalupo et al., 2026 (reference panel)
c_lp_sm_0377	NBT	1	1	33.97	Bazzicalupo et al., 2026 (reference panel)
c_lp_sm_0390	NBT	1	1	32.92	Bazzicalupo et al., 2026 (reference panel)
c_lp_sm_0452	NBT	1	1	35.17	Bazzicalupo et al., 2026 (reference panel)
c_lp_sm_0474	NBT	1	2	40.61	Bazzicalupo et al., 2026 (reference panel)
c_lp_sm_0614	NBT	1	2	53.27	Bazzicalupo et al., 2026 (reference panel)
c_lp_ca_0189	NBT	1	2	0.94	This study
c_lp_ca_0190	NBT	1	2	1.24	This study
c_lp_ca_0192	NBT	1	1	0.93	This study
c_lp_ca_0201	NBT	1	2	1.15	This study

c_lp_ca_0218	NBT	1	2	1.11	This study
c_lp_ca_0297	MIX	0.5	1	0.94	This study
c_lp_ca_0316	NBT	1	1	1.19	This study
c_lp_ca_0317	MIX	0.5	1	0.74	This study
c_lp_ca_0318	NBT	1	1	1.16	This study
c_lp_ca_0321	MIX	0.5	1	1.12	This study
c_lp_ca_0322	NBT	1	1	1.16	This study
c_lp_ca_0323	NBT	1	1	1.10	This study
c_lp_ca_0353	NBT	1	1	1.19	This study
c_lp_ca_0375	NBT	1	1	1.14	This study
c_lp_ca_0418	NBT	1	2	0.98	This study
c_lp_ca_0426	NBT	1	1	1.06	This study
c_lp_ca_0427	NBT	1	1	0.98	This study
c_lp_ca_0428	MIX	0.5	2	1.07	This study
c_lp_ca_0432	NBT	1	1	1.01	This study
c_lp_ca_0441	NBT	1	1	0.96	This study
c_lp_ca_0442	MIX	0.75	1	1.08	This study
c_lp_ca_0446	NBT	1	1	0.90	This study
c_lp_ca_0458	NBT	1	1	0.66	This study
c_lp_ca_0466	NBT	1	2	1.69	This study
c_lp_ca_0467	NBT	1	2	1.27	This study
c_lp_ca_0499	MIX	0.5	2	0.60	This study
c_lp_ca_0552	NBT	1	2	0.95	This study
c_lp_ca_0555	NBT	1	2	1.44	This study
c_lp_ca_0567	NBT	1	2	1.06	This study
c_lp_ca_0571	MIX	0.5	1	2.37	This study
c_lp_ca_0574	NBT	1	2	1.39	This study
c_lp_ca_0575	NBT	1	2	0.74	This study
c_lp_ca_0576	NBT	1	1	0.89	This study
c_lp_ca_0584	NBT	1	1	1.18	This study
c_lp_ca_0585	NBT	1	2	1.69	This study
c_lp_ca_0589	NBT	1	1	0.31	This study
c_lp_ca_0591	NBT	1	2	1.27	This study
c_lp_ca_0593	NBT	1	2	1.28	This study
c_lp_ca_0595	MIX	0.5	1	1.17	This study
c_lp_ca_0597	NBT	1	1	2.15	This study
c_lp_ca_0598	NBT	1	2	1.35	This study
c_lp_ca_0599	MIX	0.5	1	1.32	This study
c_lp_ca_0675	NBT	1	1	1.63	This study
c_lp_ca_0748	MIX	0.75	1	1.62	This study
c_lp_ca_0750	MIX	0.75	2	0.77	This study
c_lp_ca_0754	NBT	1	2	1.05	This study
c_lp_ca_0755	MIX	0.75	2	1.34	This study
c_lp_ca_0762	MIX	0.75	1	1.22	This study
c_lp_ca_0764	MIX	0.75	2	1.26	This study
c_lp_ca_0792	MIX	0.75	1	1.05	This study
c_lp_ca_0799	MIX	0.75	1	1.25	This study
c_lp_ca_0801	MIX	0.75	2	1.00	This study
c_lp_ca_0808	MIX	0.75	2	1.19	This study
c_lp_ca_0817	MIX	0.75	2	0.87	This study
c_lp_ca_0818	MIX	0.75	2	0.99	This study
c_lp_ca_0819	MIX	0.75	2	1.13	This study
c_lp_ca_0820	MIX	0.75	2	1.09	This study
c_lp_ca_0827	MIX	0.75	2	1.96	This study
c_lp_ca_0856	NBT	1	1	1.00	This study
c_lp_ca_1361	MIX	0.5	1	0.84	This study
c_lp_ca_1366	MIX	0.5	2	0.92	This study
c_lp_ca_1397	MIX	0.75	1	1.02	This study
c_lp_ca_1423	MIX	0.5	2	1.12	This study
c_lp_ca_1478	NBT	1	1	0.89	This study
c_lp_ca_1652	MIX	0.75	2	1.92	This study

c_lp_ca_1677	MIX	0.75	1	1.01	This study
c_lp_ca_1767	MIX	0.875	1	1.00	This study
c_lp_ca_1794	MIX	0.75	2	0.65	This study
c_lp_ca_1815	NBT	1	2	0.99	This study
c_lp_ca_2009	MIX	0.75	1	1.16	This study
c_lp_ca_2010	MIX	0.875	2	1.26	This study
c_lp_ca_2011	MIX	0.75	2	1.02	This study
c_lp_ca_2013	MIX	0.75	1	0.90	This study
c_lp_ca_2210	MIX	0.875	2	1.43	This study
c_lp_do_0012	BT	0	1	0.55	This study
c_lp_do_0015	BT	0	2	0.69	This study
c_lp_do_0017	BT	0	2	0.97	This study
c_lp_do_0018	BT	0	2	0.69	This study
c_lp_do_0021	BT	0	1	0.64	This study
c_lp_do_0022	BT	0	2	0.77	This study
c_lp_do_0023	BT	0	2	2.53	This study
c_lp_do_0025	BT	0	2	0.62	This study
c_lp_do_0026	BT	0	2	2.14	This study
c_lp_do_0031	BT	0	1	1.12	This study
c_lp_do_0032	BT	0	2	0.51	This study
c_lp_do_0056	BT	0	1	1.23	This study
c_lp_do_0084	BT	0	2	1.28	This study
c_lp_do_0154	BT	0	2	1.06	This study
c_lp_do_0157	BT	0	1	2.98	This study
c_lp_do_0172	BT	0	2	0.65	This study
c_lp_do_0196	BT	0	1	2.31	This study
c_lp_do_0198	BT	0	2	2.24	This study
c_lp_do_0203	BT	0	1	1.51	This study
c_lp_do_0204	BT	0	2	1.79	This study
c_lp_do_0210	BT	0	1	1.21	This study
c_lp_do_0214	BT	0	1	0.97	This study
c_lp_do_0216	BT	0	1	0.89	This study
c_lp_do_0233	BT	0	2	2.52	This study
c_lp_do_0234	BT	0	2	1.68	This study
c_lp_do_0247	BT	0	1	1.60	This study
c_lp_do_0249	BT	0	2	1.25	This study
c_lp_do_0251	BT	0	1	1.87	This study
c_lp_do_0253	BT	0	2	1.02	This study
c_lp_do_0254	BT	0	2	1.25	This study
c_lp_do_0262	BT	0	1	0.53	This study
c_lp_do_0263	BT	0	2	1.97	This study
c_lp_do_0275	BT	0	2	1.02	This study
c_lp_do_0279	BT	0	2	1.49	This study
c_lp_do_0280	BT	0	2	1.40	This study
c_lp_do_0315	BT	0	2	1.33	This study
c_lp_do_0337	NBT	1	1	2.07	This study
c_lp_do_0381	MIX	0.5	2	2.82	This study
c_lp_do_0383	BT	0	1	0.92	This study
c_lp_do_0386	MIX	0.5	2	0.29	This study
c_lp_do_0392	BT	0	2	1.87	This study
c_lp_do_0449	BT	0	1	2.97	This study
c_lp_do_0457	NBT	1	2	1.02	This study
c_lp_do_0462	BT	0	1	2.48	This study
c_lp_do_0464	BT	0	2	0.55	This study
c_lp_do_0465	BT	0	2	0.51	This study
c_lp_do_0486	MIX	0.25	2	0.85	This study
c_lp_do_0487	BT	0	2	1.44	This study
c_lp_do_0534	MIX	0.5	1	1.44	This study
c_lp_do_0536	MIX	0.25	1	0.75	This study
c_lp_do_0606	MIX	0.25	2	1.12	This study
c_lp_do_0608	MIX	0.25	2	0.87	This study

c_lp_do_0612	MIX	0.75	2	0.90	This study
c_lp_do_0627	MIX	0.25	1	0.92	This study
c_lp_do_0681	MIX	0.5	1	0.87	This study
c_lp_do_0682	BT	0	2	0.26	This study
c_lp_do_0776	BT	0	1	1.84	This study
c_lp_do_0789	MIX	0.75	2	2.61	This study
c_lp_do_1347	MIX	0.25	1	0.78	This study
c_lp_do_1348	BT	0	2	0.75	This study
c_lp_do_1416	BT	0	1	0.76	This study
c_lp_do_1418	MIX	0.438	1	0.64	This study
c_lp_do_1482	MIX	0.5	2	1.12	This study
c_lp_do_1496	NBT	1	2	1.25	This study
c_lp_do_1798	MIX	0.125	2	1.22	This study
c_lp_do_1799	BT	0.25	2	1.45	This study
c_lp_do_1843	MIX	0.125	1	1.60	This study
c_lp_do_1844	MIX	0.25	1	1.53	This study
c_lp_do_1845	BT	0	1	1.17	This study
c_lp_do_1847	BT	0	1	1.72	This study
c_lp_do_1852	MIX	0.125	2	1.67	This study
c_lp_do_1853	MIX	0.25	2	1.44	This study
c_lp_do_1854	MIX	0.625	2	1.05	This study
c_lp_do_1865	BT	0	2	0.86	This study
c_lp_do_1866	MIX	0.25	1	1.76	This study
c_lp_do_2280	MIX	0.25	1	0.70	This study
c_lp_sm_0274	NBT	1	1	1.31	This study
c_lp_do_0003	BT	0	2	0.15	This study
c_lp_do_0050	BT	0	2	0.25	This study
c_lp_do_0085	BT	0	2	0.25	This study
c_lp_do_0187	BT	0	1	0.46	This study
c_lp_do_0255	BT	0	1	0.24	This study
c_lp_do_0291	BT	0	1	0.34	This study
c_lp_do_0535	BT	0	1	0.04	This study
c_lp_do_0538	BT	0	1	0.33	This study
c_lp_do_0605	MIX	0.5	2	0.50	This study
c_lp_do_0607	MIX	0.25	1	0.09	This study
c_lp_do_0609	BT	0	2	0.30	This study
c_lp_do_0793	BT	0	1	0.13	This study
c_lp_do_1417	BT	0	1	0.20	This study
c_lp_do_1850	BT	0	1	0.30	This study
c_lp_do_1868	BT	0	1	0.14	This study
c_lp_do_2145	BT	0	1	0.28	This study
c_lp_sm_0136	NBT	1	1	0.91	This study
c_lp_do_0007	BT	0	1	21.42	Abascal et al., 2016
c_lp_do_0153	BT	0	1	25.49	Abascal et al., 2016
c_lp_do_0173	BT	0	1	26.46	Abascal et al., 2016
c_lp_do_0443	BT	0	1	27.40	Abascal et al., 2016
c_lp_sm_0138	NBT	1	1	26.45	Abascal et al., 2016
c_lp_sm_0140	NBT	1	1	25.16	Abascal et al., 2016
c_lp_sm_0185	NBT	1	1	25.40	Abascal et al., 2016
c_lp_sm_0186	NBT	1	1	26.69	Abascal et al., 2016
c_lp_sm_0221	NBT	1	1	30.49	Abascal et al., 2016
c_lp_sm_0298	NBT	1	1	29.25	Abascal et al., 2016
c_lp_sm_0359	NBT	1	1	27.11	Abascal et al., 2016
c_lp_do_0141	BT	0	1	4.92	Lucena-Pérez et al., 2021
c_lp_do_0144	BT	0	2	6.00	Lucena-Pérez et al., 2021
c_lp_do_0162	BT	0	2	5.80	Lucena-Pérez et al., 2021
c_lp_do_0163	BT	0	2	5.52	Lucena-Pérez et al., 2021
c_lp_do_0300	BT	0	1	5.58	Lucena-Pérez et al., 2021
c_lp_do_0333	BT	0	2	6.10	Lucena-Pérez et al., 2021
c_lp_do_0335	BT	0	2	5.16	Lucena-Pérez et al., 2021
c_lp_do_0444	BT	0	2	5.24	Lucena-Pérez et al., 2021

c_lp_sm_0134	NBT	1	2	5.80	Lucena-Pérez et al., 2021
c_lp_sm_0155	NBT	1	2	5.46	Lucena-Pérez et al., 2021
c_lp_sm_0156	NBT	1	2	5.95	Lucena-Pérez et al., 2021
c_lp_sm_0161	NBT	1	2	5.19	Lucena-Pérez et al., 2021
c_lp_sm_0206	NBT	1	2	5.62	Lucena-Pérez et al., 2021
c_lp_sm_0208	NBT	1	2	5.29	Lucena-Pérez et al., 2021
c_lp_sm_0213	NBT	1	2	5.41	Lucena-Pérez et al., 2021
c_lp_sm_0226	NBT	1	1	5.56	Lucena-Pérez et al., 2021
c_lp_sm_0276	NBT	1	2	5.51	Lucena-Pérez et al., 2021
c_lp_sm_0320	NBT	1	2	5.53	Lucena-Pérez et al., 2021
c_lp_sm_0325	NBT	1	2	5.35	Lucena-Pérez et al., 2021
c_lp_sm_0450	NBT	1	1	5.46	Lucena-Pérez et al., 2021

Table S2. Effect sizes of the differences in demographic trajectories inferred from imputed and high-coverage datasets. Cohen's *d* effect sizes (95% CI) comparing effective population size (N_e) estimates inferred from imputed datasets at four imputation accuracy levels (R1–R4, from lower to higher concordance) against the high-coverage dataset for the last 100 generations for the BT (n = 14) and NBT (n = 14) populations. N_e estimates were obtained with GONE, and effect sizes were computed separately for recent (1–15 generations), old (16–100 generations) and overall demographic trajectories, showing that most differences between the demographic trajectories inferred high-coverage and the imputed datasets come from older periods of time.

Cohen's *d* [%95 CI]		1-15 generations ago	16-100 generations ago	All
BT	R1	0.46 [-0.30, 1.22]	0.77 [0.46, 1.09]	0.62 [0.34, 0.91]
	R2	-0.04 [-0.79, 0.71]	0.37 [0.06, 0.67]	0.26 [-0.02, 0.54]
	R3	-0.09 [-0.83, 0.66]	0.24 [-0.06, 0.55]	0.16 [-0.12, 0.44]
	R4	-0.22 [-0.97, 0.53]	0.19 [-0.11, 0.49]	0.13 [-0.15, 0.41]
NBT	R1	0.01 [-0.74, 0.76]	1.48 [1.14, 1.83]	0.20 [-0.08, 0.48]
	R2	0.02 [-0.73, 0.77]	0.38 [0.08, 0.69]	0.06 [-0.22, 0.33]
	R3	-0.00 [-0.75, 0.75]	-0.10 [-0.41, 0.20]	-0.02 [-0.29, 0.26]
	R4	0.03 [-0.71, 0.78]	0.74 [0.42, 1.05]	0.10 [-0.18, 0.38]

Table S3. Effect sizes of differences in inbreeding coefficients (F_{ROH}) between imputed and high-coverage datasets across different HBD classes. Cohen's *d* effect sizes (95% CI) and median differences for F_{ROH} estimates obtained from imputed datasets at four imputation accuracy levels (R1–R4, from lower to higher concordance) relative to those derived from the high-coverage dataset. Inbreeding coefficients were estimated across different HBD classes, reflecting inbreeding from different temporal depths. Overall, F_{ROH} estimates from imputed data tended to be inflated compared to high-coverage data, with larger deviations observed at lower imputation accuracy levels and for older inbreeding signals (e.g., HBD class = 1024).

	HBD class 128		HBD class 256		HBD class 1024	
	Cohen's *d* [%95 CI]	Median ΔF_{ROH}	Cohen's *d* [%95 CI]	Median ΔF_{ROH}	Cohen's *d* [%95 CI]	Median ΔF_{ROH}
R1	0.085 [0.064, 0.107]	0.015	0.232 [0.205, 0.260]	0.079	0.628 [0.534, 0.722]	0.140
R2	0.068 [0.053, 0.083]	0.012	0.171 [0.150, 0.191]	0.052	0.404 [0.344, 0.463]	0.106
R3	0.100 [0.085, 0.115]	0.016	0.120 [0.105, 0.135]	0.022	0.271 [0.230, 0.311]	0.068
R4	0.062 [0.050, 0.075]	0.010	0.080 [0.066, 0.094]	0.014	0.213 [0.180, 0.247]	0.059

Table S4. Effect sizes of differences in ADMIXTURE ancestry proportions inferred from imputed and high-coverage datasets. Cohen's *d* effect sizes (95% CI) and median differences comparing ADMIXTURE ancestry proportions estimated for admixed individuals (Q_{MIX}) from imputed datasets at four imputation accuracy levels (R1–R4, from lower to higher concordance) against those obtained from the high-coverage dataset. Differences in ancestry proportion estimates were negligible across all imputation accuracy levels.

Accuracy level	Cohen's *d* [%95 CI]	Median ΔQ_{MIX}
R1	0.0014 [-0.0021, 0.0050]	0.0003
R2	0.0023 [0.0000, 0.0047]	0.0004
R3	0.0031 [0.0010, 0.0052]	0.0008
R4	0.0006 [-0.0012, 0.0025]	0.0004

Table S5. Mean confusion matrices of admixed individuals with majority of BT pedigree-expected ancestry ($0 < \theta_s < 0.5$). Cells show the proportion of SNPs assigned to each ancestry class in the imputed (columns) compared to the high-coverage dataset (rows). R1 to R4 represent the four imputation accuracy levels tested, from lower to higher concordance. Values in parentheses indicate standard deviations across individuals. Bolded values highlight the assigned proportions matching between both datasets.

Most-BT ($0 < \theta_s < 0.5$)												
Imputed / High-cov	BT-BT				BT-NBT				NBT-NBT			
	R1	R2	R3	R4	R1	R2	R3	R4	R1	R2	R3	R4
BT-BT	**0.998 (0.002)**	**0.999 (0.001)**	**0.996 (0.001)**	**0.998 (0.001)**	0.002 (0.002)	0.001 (0.001)	0.003 (0.002)	0.002 (0.002)	0.000 (0.001)	0.000 (0.000)	0.000 (0.001)	0.000 (0.001)
BT-NBT	0.051 (0.010)	0.025 (0.008)	0.013 (0.004)	0.008 (0.003)	**0.927 (0.013)**	**0.968 (0.010)**	**0.983 (0.005)**	**0.990 (0.004)**	0.021 (0.007)	0.008 (0.005)	0.004 (0.003)	0.002 (0.002)
NBT-NBT	0.000 (0.000)	0.000 (0.000)	0.000 (0.000)	0.000 (0.000)	0.384 (0.468)	0.095 (0.132)	0.313 (0.467)	0.384 (0.469)	**0.616 (0.468)**	**0.905 (0.132)**	**0.687 (0.467)**	**0.616 (0.469)**

Table S6. Mean confusion matrices of admixed individuals with majority of NBT pedigree-expected ancestry ($0.5 < \theta_s < 1$). Proportion of SNPs assigned to each ancestry class in the imputed data across coverages (columns) compared to the high-coverage data (rows). R1 to R4 represent the four imputation accuracy levels tested, from lower to higher concordance. Values in parentheses indicate standard deviations across individuals. Bolded values highlight the assigned proportions matching between both datasets.

Most-NBT ($0.5 < \theta_s < 1$)												
Imputed / High-cov	BT-BT				BT-NBT				NBT-NBT			
	R1	R2	R3	R4	R1	R2	R3	R4	R1	R2	R3	R4
BT-BT	**0.927 (0.072)**	**0.935 (0.082)**	**0.927 (0.076)**	**0.929 (0.080)**	0.073 (0.072)	0.065 (0.082)	0.073 (0.076)	0.071 (0.080)	0.000 (0.000)	0.000 (0.000)	0.000 (0.000)	0.000 (0.000)
BT-NBT	0.034 (0.007)	0.017 (0.005)	0.007 (0.004)	0.003 (0.002)	**0.928 (0.017)**	**0.963 (0.007)**	**0.983 (0.007)**	**0.991 (0.005)**	0.038 (0.013)	0.019 (0.006)	0.004 (0.004)	0.004 (0.003)
NBT-NBT	0.001 (0.002)	0.001 (0.001)	0.001 (0.001)	0.000 (0.000)	0.007 (0.003)	0.006 (0.005)	0.007 (0.004)	0.006 (0.006)	**0.992 (0.005)**	**0.994 (0.005)**	**0.995 (0.004)**	**0.995 (0.005)**

Table S7. Mean confusion matrix of admixed individuals with the equal proportions of BT and NBT pedigree-expected ancestry (θ_s = 0.5). Proportion of SNPs assigned to each ancestry class in the imputed data (columns) compared to the high-coverage data (rows). R1 to R4 represent the four imputation accuracy levels tested, from lower to higher concordance. Values in parentheses indicate standard deviations across individuals. Bolded values highlight the assigned proportions matching between both datasets.

BT≈NBT (θ_s = 0.5)												
Imputed / High-cov	BT-BT				BT-NBT				NBT-NBT			
	R1	R2	R3	R4	R1	R2	R3	R4	R1	R2	R3	R4
BT-BT	**0.981 (0.020)**	**0.972 (0.034)**	**0.967 (0.041)**	**0.937 (0.062)**	0.019 (0.020)	0.028 (0.034)	0.033 (0.041)	0.063 (0.062)	0.000 (0.000)	0.000 (0.000)	0.000 (0.000)	0.000 (0.000)
BT-NBT	0.049 (0.006)	0.022 (0.003)	0.006 (0.003)	0.004 (0.001)	**0.925 (0.010)**	**0.969 (0.007)**	**0.991 (0.003)**	**0.996 (0.002)**	0.026 (0.006)	0.009 (0.005)	0.003 (0.001)	0.001 (0.001)
NBT-NBT	0.001 (0.003)	0.000 (0.000)	0.000 (0.000)	0.000 (0.000)	0.066 (0.075)	0.107 (0.199)	0.223 (0.278)	0.159 (0.303)	**0.932 (0.077)**	**0.893 (0.199)**	**0.776 (0.278)**	**0.841 (0.303)**

SUPPLEMENTARY FIGURES

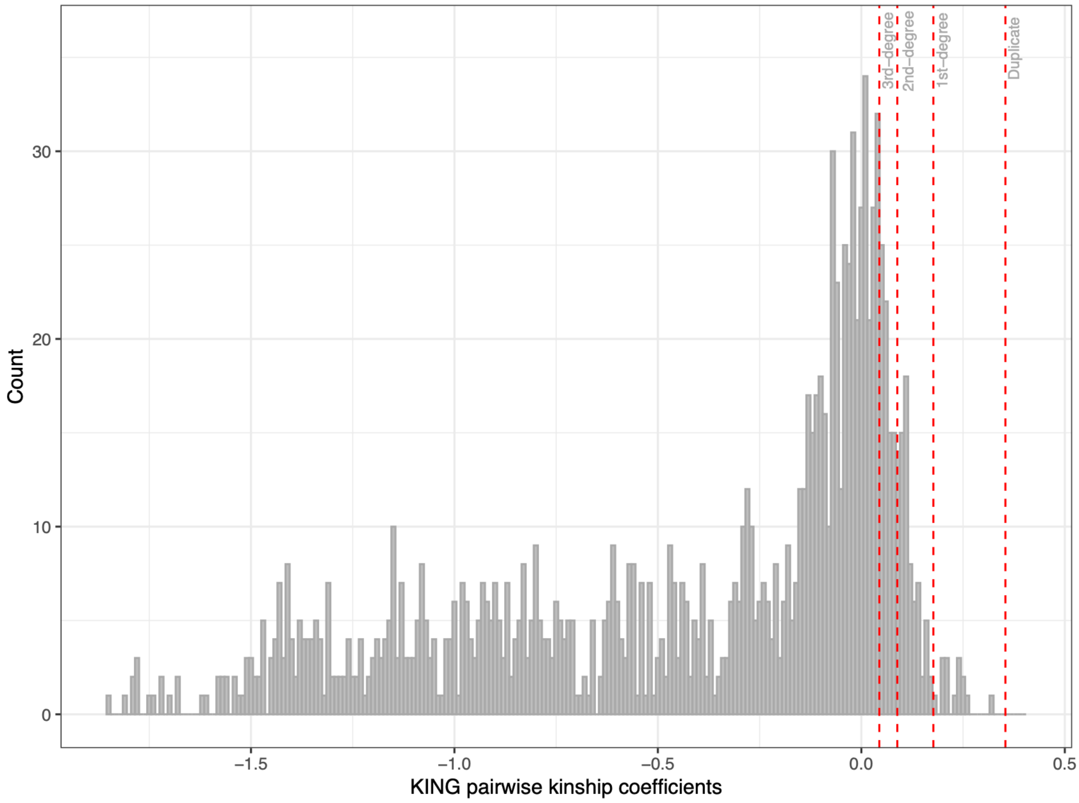

Figure S1. Distribution of pairwise kinship coefficients estimated with KING among reference panel individuals. Red vertical lines indicate the theoretical KING threshold values used to classify relatedness categories; from right to left, these correspond to duplicate/monozygotic twins (>0.354), first-degree relatives (0.177, 0.354), second-degree relatives (0.0884, 0.177), and third-degree relatives (0.0442, 0.0884).

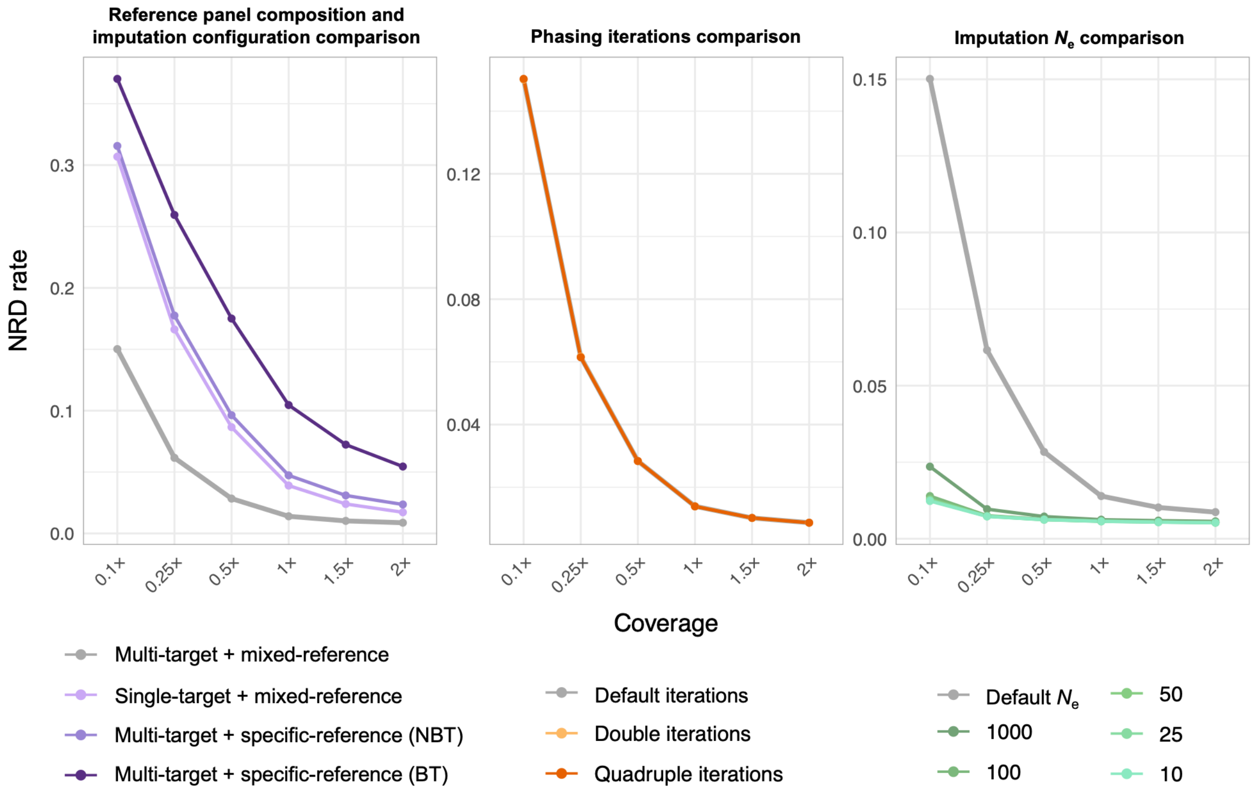

Figure S2. Non-reference discordance (NRD) rates across imputation assays. Left panel summarizes NRD rates obtained under alternative reference panel compositions — either a mixed-reference (including all high-coverage sequenced individuals) or a specific-reference (including only individuals from the bottlenecked, BT, or non-bottlenecked, NBT, populations) — and different imputation configurations, namely single-target imputation (only the downsampled individual was imputed) and multi-target imputation (the downsampled individual was imputed jointly with additional whole-genome sequenced samples sharing the same ancestral background). The center panel shows NRD rates under the multi-target, mixed-reference imputation assay (which yielded the highest overall accuracy) across different settings of the SHAPEIT –mcmc parameter (phasing iterations). The right panel displays NRD rates for the same assay under varying effective population sizes (GLIMPSE_phase –ne parameter) during imputation. Increasing the number of phasing iterations did not lead to further improvements in imputation accuracy, whereas reducing the assumed effective population size resulted in a substantial decrease in NRD rates.

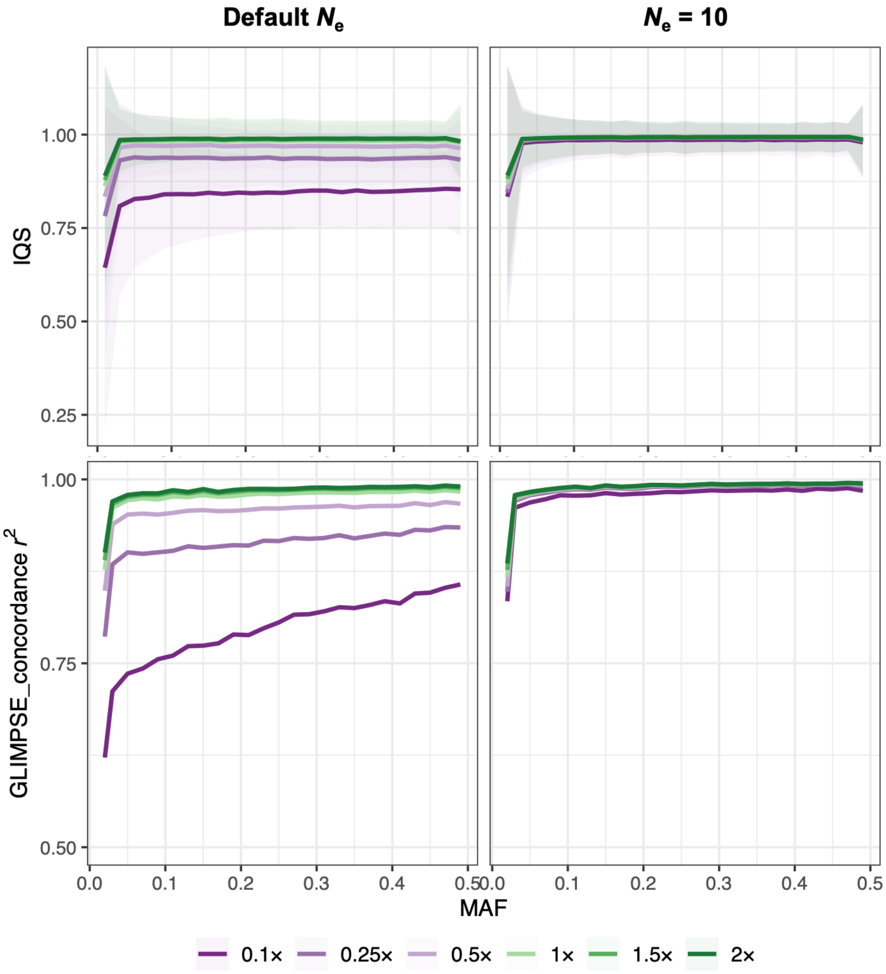

Figure S3. Imputation accuracy across minor allele frequency (MAF) bins and sequencing coverage levels. Accuracy was evaluated in leave-one-out imputation assays within a multi-target framework using a mixed-ancestry reference panel. It is reported as the imputation quality score (IQS; top) and r^2 (bottom), comparing results obtained with the GLIMPSE imputation –ne parameter set to its default value (left) or to 10 (right). For IQS, mean values across MAF bins are shown as thick lines, with shaded regions indicating ±1 standard deviation. The results illustrate a decline in imputation accuracy for low-frequency variants, as well as a marked improvement when an appropriate effective population size parameter is specified.

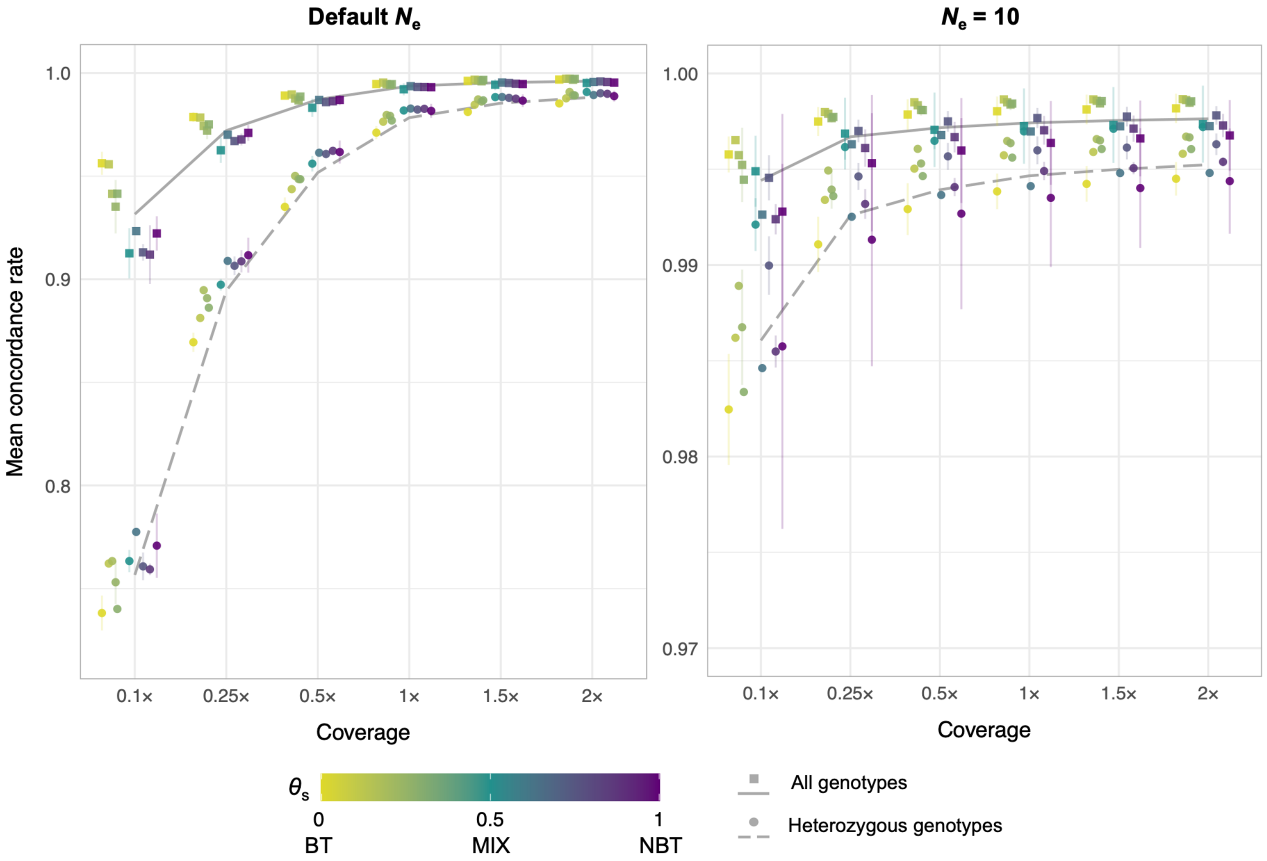

Figure S4. Per-individual genotype concordance between imputed and high-coverage datasets. Imputation was performed within the multi-target configuration with a mixed-ancestry reference panel framework, under default GLIMPSE imputation –ne parameter (left) and –ne set to 10 (right). Mean genotype concordance rate (± SD) was calculated as the proportion of correctly imputed genotypes relative to high-coverage genotypes in each sample. The solid line indicates the mean concordance across individuals and coverage levels when considering all genotypes, whereas the dashed line shows concordance calculated using only heterozygous genotypes. Squares represent mean concordance values grouped by θ_s, a pedigree-based ancestry coefficient ranging from pure BT ancestry (θ_s = 0; green) to pure NBT ancestry (θ_s = 1; purple). Circles denote concordance values calculated from heterozygous genotypes only. Concordance restricted to heterozygous sites is consistently lower than overall concordance, increasing with higher NBT ancestry, especially under default imputation –ne.

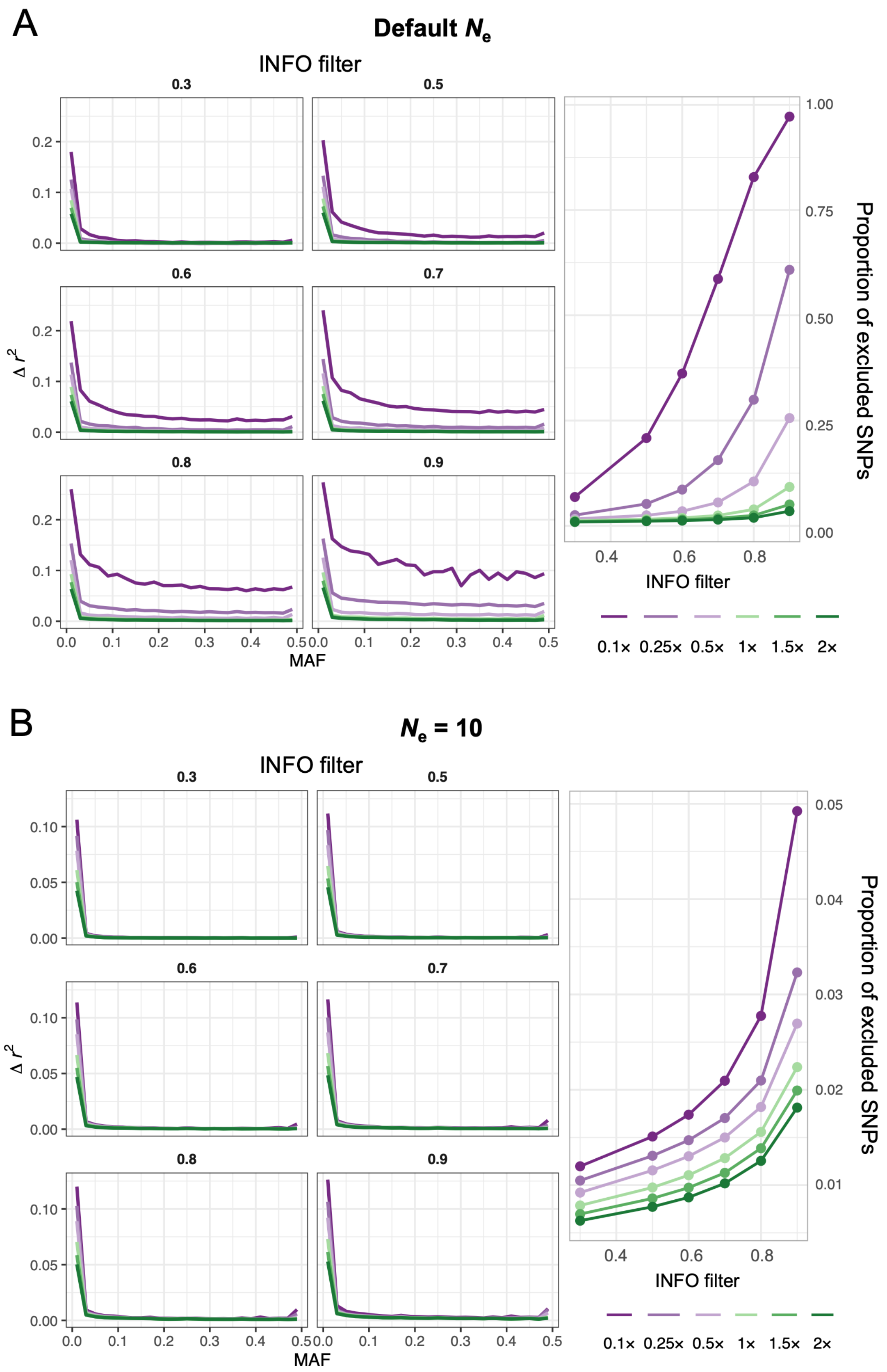

Figure S5. Effect of INFO filtering on imputation accuracy and marker retention when imputing with default GLIMPSE –ne parameter (A) or with –ne set to 10 (B). Left panels show the difference of imputation r^2 per minor allele frequency (MAF) bin after filtering SNPs using increasing INFO score thresholds (INFO > 0.3 to INFO > 0.9). Right panels display the proportion of SNPs removed at each coverage level under each INFO threshold.

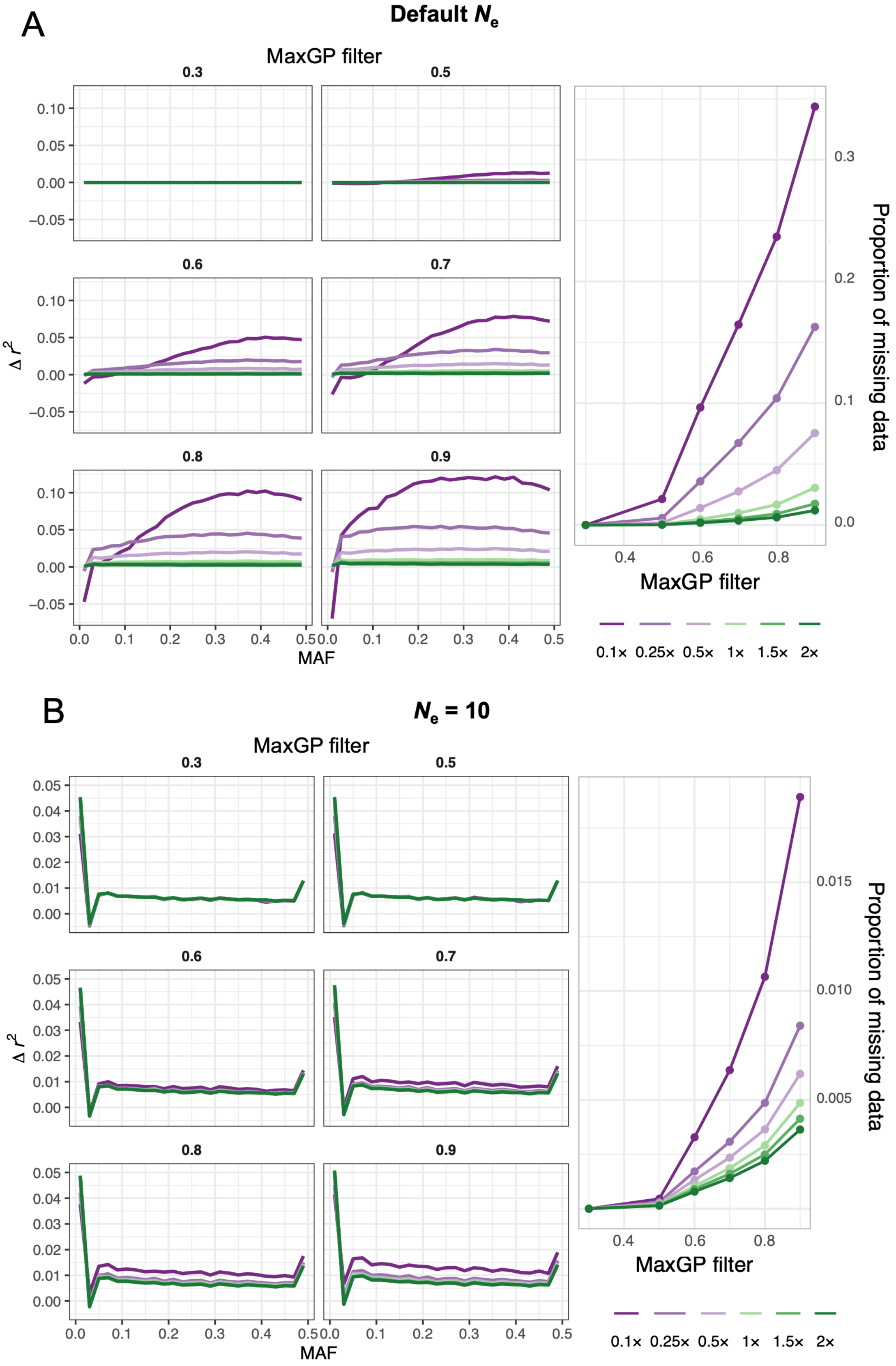

Figure S6. Effect of maximum genotype probability filtering on imputation accuracy and missing data generation when imputing with GLIMPSE default –ne parameter (A) or with –ne set to 10 (B). Left panels show the difference of imputation r^2 per minor allele frequency (MAF) bin after filtering genotypes using increasing maximum genotype probability thresholds (maxGP>0.3 to maxGP>0.9). Right panel displays the proportion of missing genotypes introduced under each maxGP threshold.

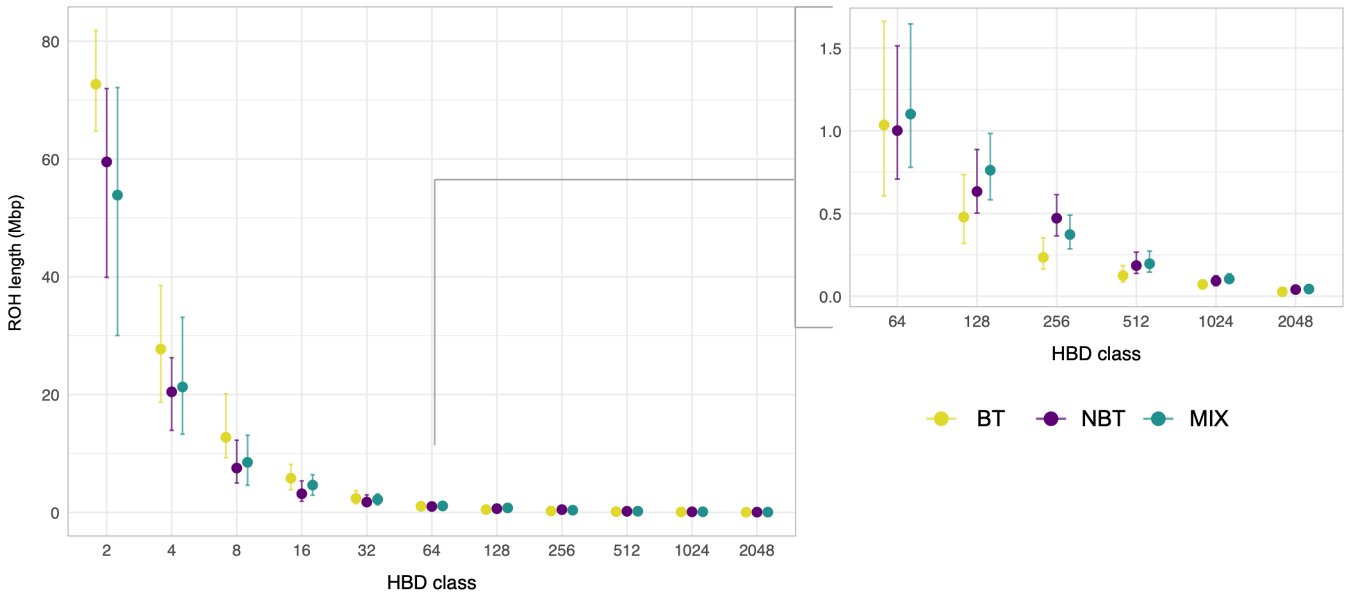

Figure S7. Mean ROH length per HBD class in the high-coverage dataset. Values are shown for HBD classes defined by RZooRoH in the BT, NBT and MIX populations, using high-coverage genotypes. Error bars represent the 25th and 75th percentiles across individuals.

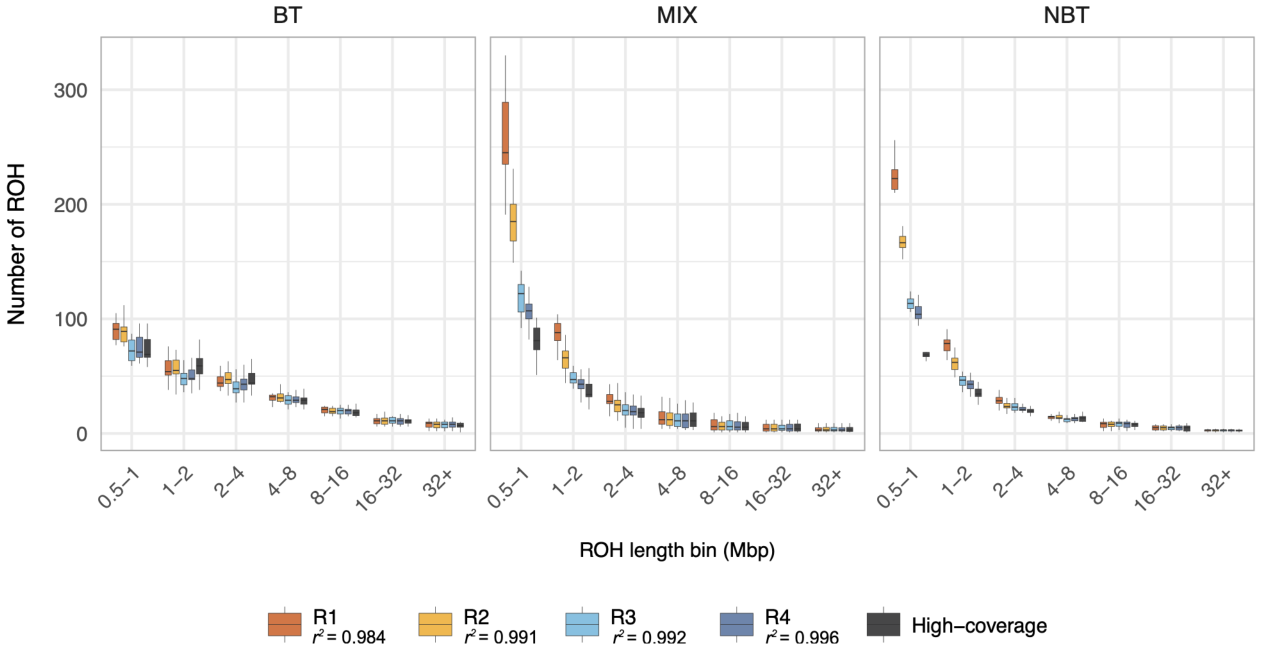

Figure S8. Number of ROH per individual across ROH length bins. Mean values are shown across ROH length bins, for each population and imputation accuracy level (R1-R4, from lower to higher concordance).

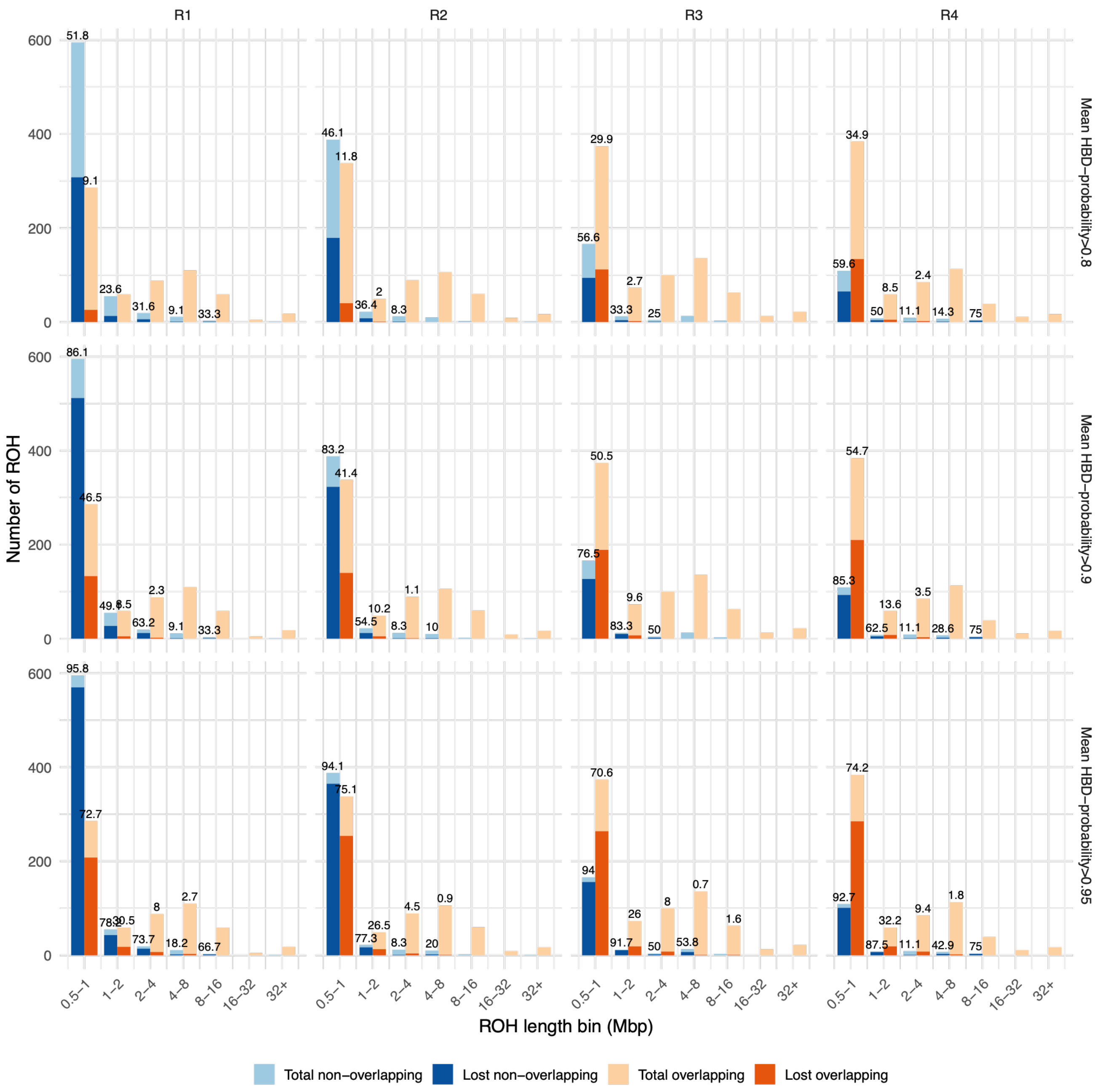

Figure S9. Effect of mean HBD-probability filtering on overlapping and non-overlapping ROH retention. Counts of overlapping and non-overlapping ROH across length bins after applying increasing mean HBD-probability thresholds. Bars show the number of ROH per bin and imputation accuracy level (R1-R4, from lower to higher concordance), with lighter colors indicating the total ROH and darker colors indicating ROH removed by filtering. Blue and orange bars represent non-overlapping and overlapping ROH, respectively. Values above bars indicate the percentage of ROH removed within each category.

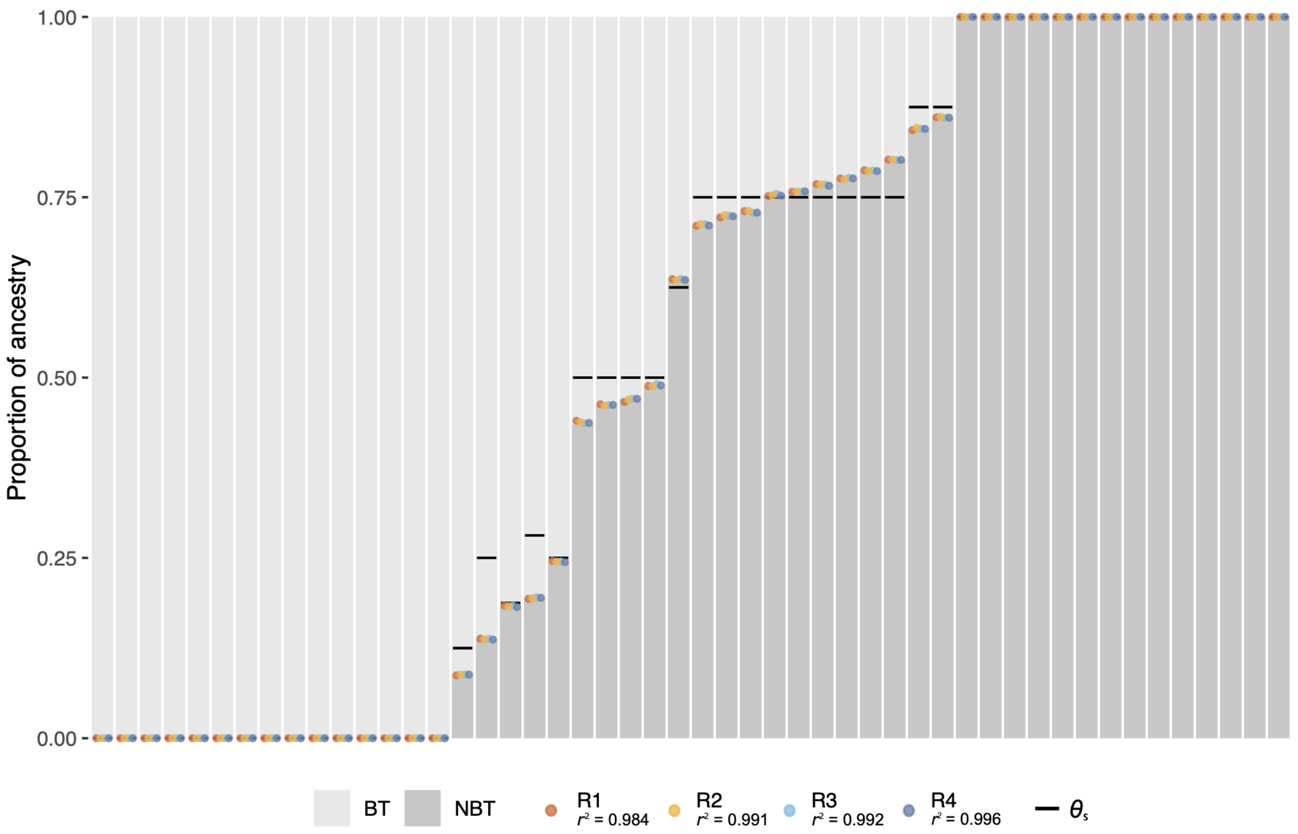

Figure S10. Ancestry proportions inferred with ADMIXTURE. Each bar represents one individual, partitioned into two ancestral components as inferred with the high-coverage dataset (BT in light gray and NBT in darker gray). Individuals are sorted by pedigree-based ancestry (θ_s, black line). Colored dots represent the ancestry proportions infered with the imputed datasets at different imputation accuracy levels (R1-R4, from lower to higher concordance).

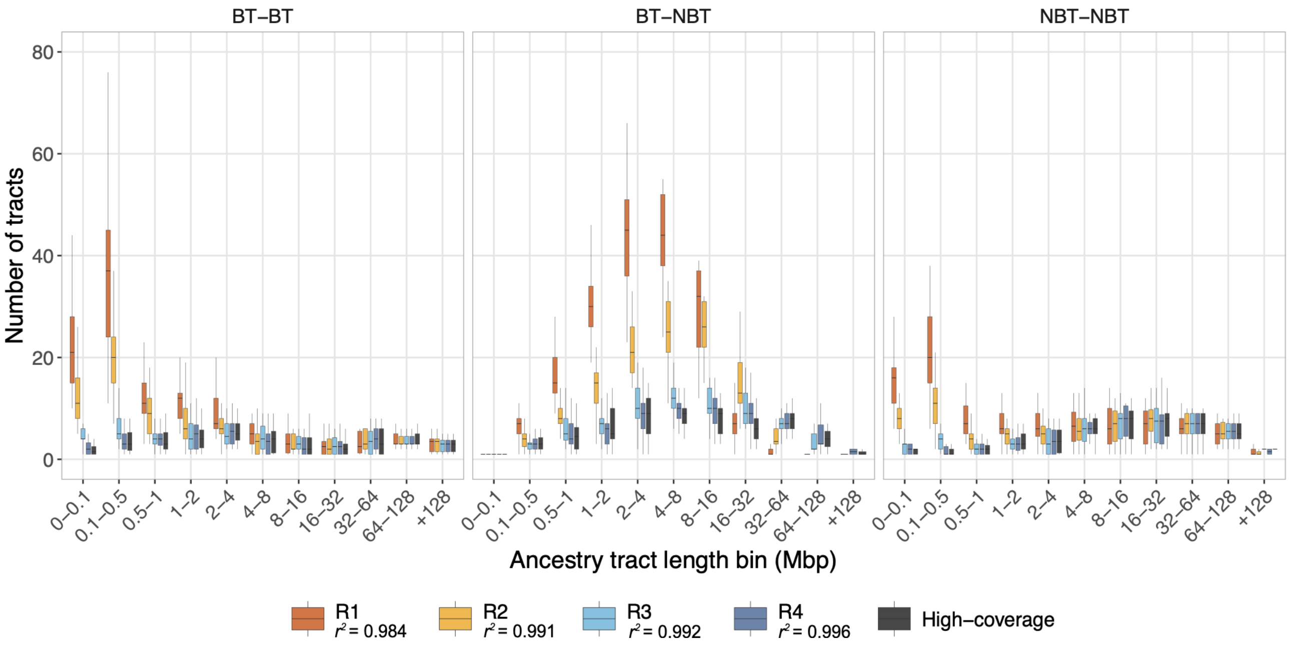

Figure S11. Distribution of ancestry tract counts across tract length bins. Number of ancestry-specific tracts per individual across tract length bins inferred with RFMix2 from high-coverage and imputed datasets with different imputation accuracy levels (R1-R4, from lower to higher concordance). Panels show homozygous BT ancestry tracts (left), heterozygous tracts (center) and homozygous NBT ancestry tracts (right).

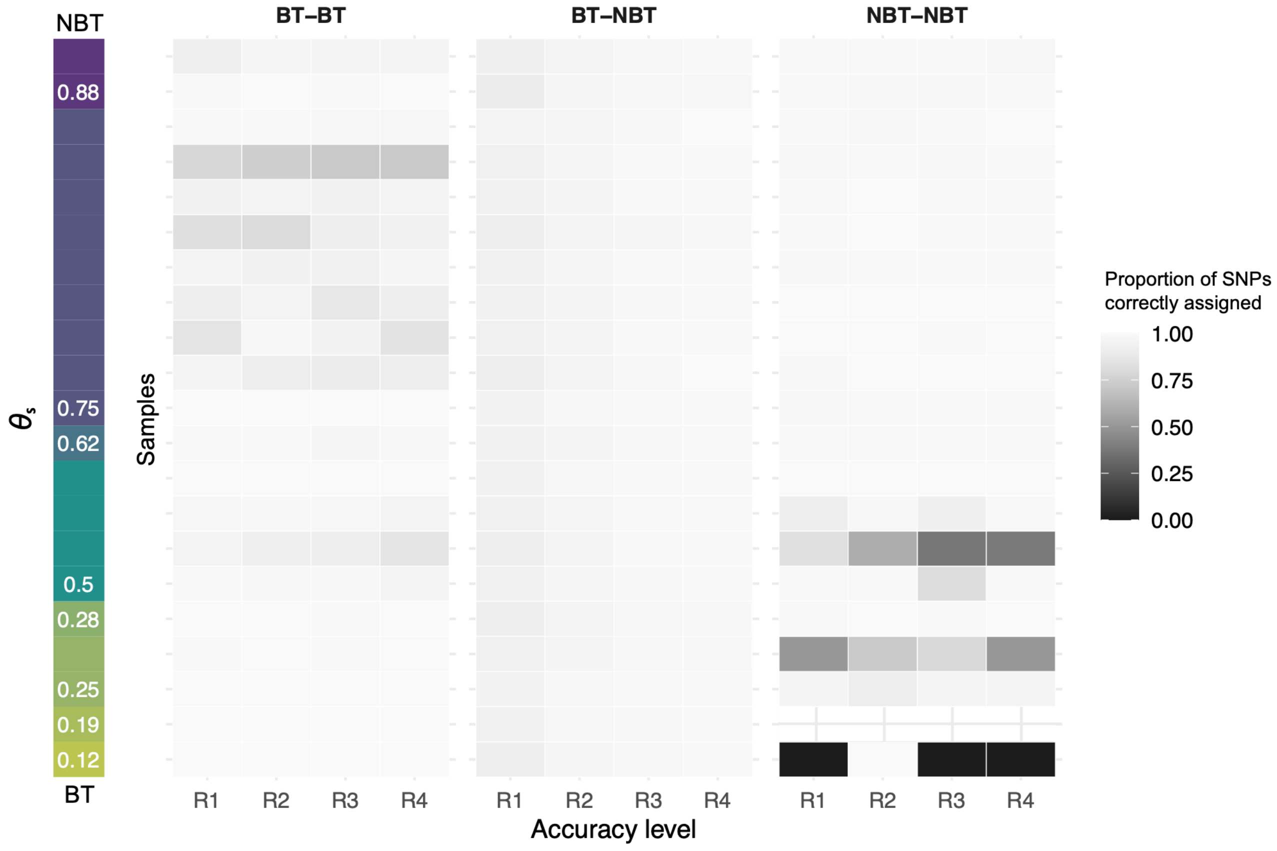

Figure S12. Accuracy of ancestry assignment in admixed individuals. Diagonal values of individual ancestry confusion matrices for admixed samples, representing the proportion of SNPs assigned to the same ancestry in both high-coverage and imputed datasets across different imputation accuracy levels (R1-R4, from lower to higher accuracy). Each row corresponds to one individual. Tile shading reflects the proportion of SNPs correctly assigned to their ancestry category (darker colors indicate lower proportion). Individuals are ordered by their expected ancestry proportions (θ_s), from BT (θ_s = 0; green) to NBT (θ_s = 1; purple). Uncolored tiles represent one that had no segments detected for the NBT-NBT ancestry in the high-coverage dataset.

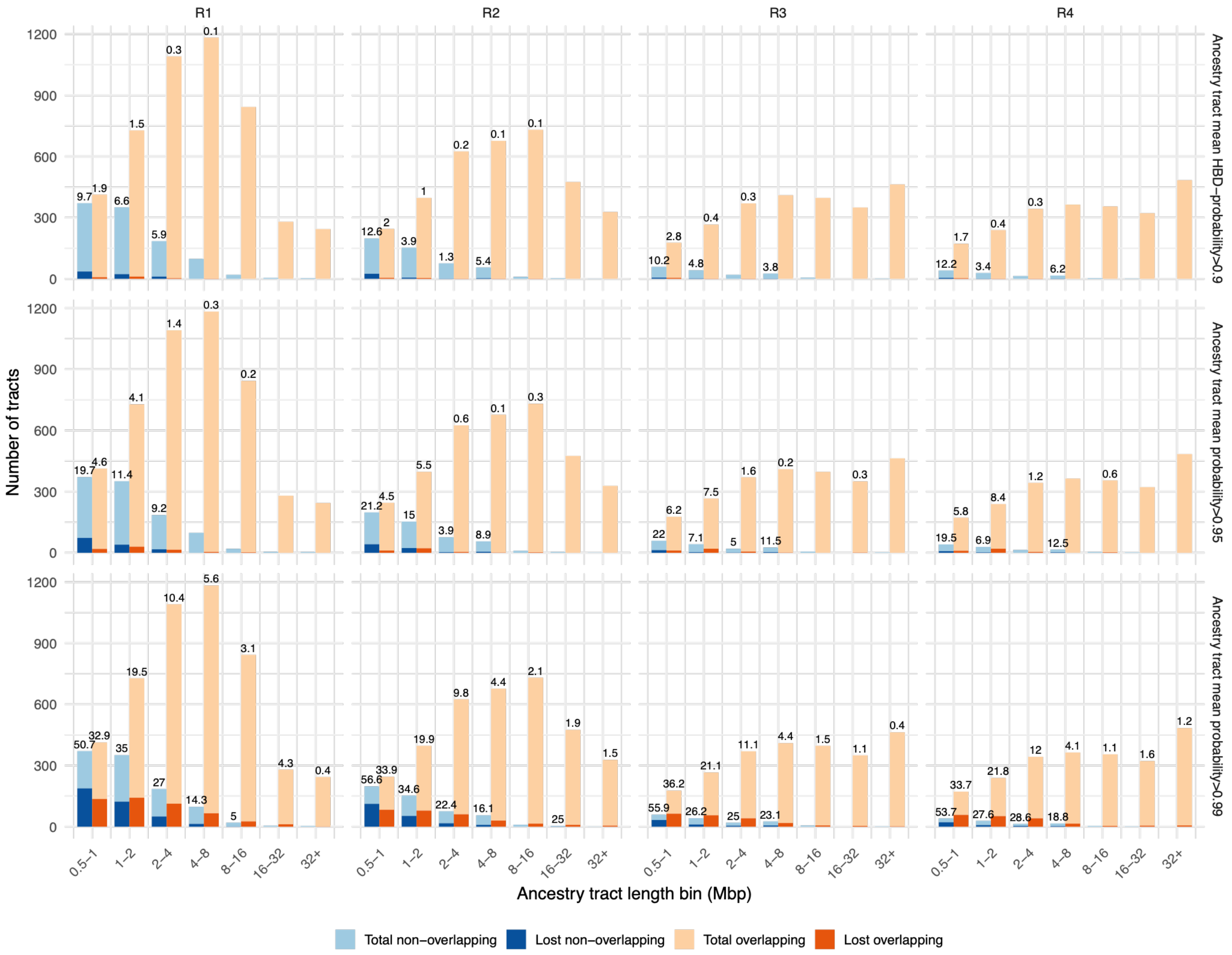

Figure S11. Effect of ancestry-assignment probability filtering on overlapping and non-overlapping ancestry tracts. Counts of overlapping and non-overlapping ancestry tracts across length bins after applying increasing mean ancestry-assignment probability thresholds. Bars show the number of tracts per bin and imputation accuracy level (R1-R4, from lower to higher accuracy), with lighter colors indicating total tracts and darker colors indicating tracts removed by filtering. Blue and orange bars represent non-overlapping and overlapping tracts, respectively. Values above bars indicate the percentage of tracts removed within each category.

REFERENCES

Abascal, F., Corvelo, A., Cruz, F., Villanueva-Cañas, J. L., Vlasova, A., Marcet-Houben, M., Martínez-Cruz, B., Cheng, J. Y., Prieto, P., Quesada, V., Quilez, J., Li, G., García, F., Rubio-Camarillo, M., Frias, L., Ribeca, P., Capella-Gutiérrez, S., Rodríguez, J. M., Câmara, F., … Godoy, J. A. (2016). Extreme genomic erosion after recurrent demographic bottlenecks in the highly endangered Iberian lynx. *Genome Biology, 17*(1), 251. https://doi.org/10.1186/s13059-016-1090-1

Bazzicalupo, E., Lorenzo-Fernández, L., Mayor-Fidalgo, L., Soriano, L., Schrider, D. R., & Godoy, J. A. (2026). Deep learning reveals genomic regions introgressed between two recurrently hybridizing lynx species. *Molecular Biology and Evolution, 43*(4), msag086. https://doi.org/10.1093/molbev/msag086

Lucena-Perez, M., Kleinman-Ruiz, D., Marmesat, E., Saveljev, A. P., Schmidt, K., & Godoy, J. A. (2021). Bottleneck-associated changes in the genomic landscape of genetic diversity in wild lynx populations. *Evolutionary Applications, 14*(11), 2664–2679. https://doi.org/10.1111/eva.13302
